# Ultrafast Lasers for Surface Texturing: Transforming the Future of Dental Biomaterials

**DOI:** 10.1021/acsabm.5c01829

**Published:** 2025-12-30

**Authors:** Anjali K K, Kishore Ginjupalli, Runki Saran, Sajan D. George, Unnikrishnan V K

**Affiliations:** † Manipal Institute of Applied Physics (MIAP), 76793 Manipal Academy of Higher Education, Manipal, Karnataka 576104, India; ‡ Department of Dental Materials, Manipal College of Dental Sciences, Manipal, 76793 Manipal Academy of Higher Education, Manipal, Karnataka 576104, India; § Centre for Applied Nanosciences (CAN), Manipal Institute of Applied Physics, 76793 Manipal Academy of Higher Education, Manipal, Karnataka 576104, India

**Keywords:** ultrafast laser, surface texturing, implants, osseointegration, microbial adhesion

## Abstract

The advancements in laser technology have enabled its widespread application in the materials science, manufacturing, and healthcare industries. Among these, laser-assisted surface modification of biomedical materials is currently one of the most widely investigated research areas owing to the multiple advantages of laser-based techniques over conventional methods. When a laser beam of adequate energy is irradiated onto a substrate, it induces ablation through melting, evaporation, and resolidification, resulting in micro/nanolevel surface features. Such laser-assisted surface treatment, a noncontact method, offers significant control over process parameters, enabling high reproducibility of surface features. Pulsed lasers, more particularly those with nanosecond, picosecond, and femtosecond pulse durations, are extensively used for surface modification of dental biomaterials due to their precision and effectiveness. The applications of laser-based surface texturing span from enhancing osseointegration and antimicrobial properties to improving tribological properties such as reduced friction, wear, and corrosion resistance. Among these, most of the research focused on the effect of laser patterning on improving the surface characteristics of biomaterials. Achieving surfaces with optimum characteristics requires an intricate understanding of laser-matter interaction mechanisms, material properties, and the effect of laser patterning parameters on the material surface. In this regard, there is considerable scope for exploring the suitability of lasers as a potential tool for surface modification of biomaterials. In addition, in-depth research is anticipated to advance the efficiency of dental biomaterials. This review aims to dive deep into the principles of controlled laser ablation, the effects of various laser parameters on material surface properties, and the role of material surface properties, on the overall performance of dental biomaterials. While laser patterning is used to alter the surface characteristics for a variety of applications, the current review focuses primarily on laser surface modification to achieve superior osseointegration and reduce microbial adhesion.

## Graphical abstract

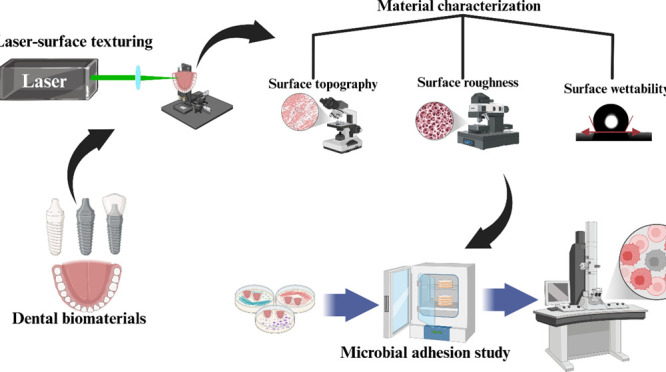

## 1 Introduction

The ability of lasers to direct a large amount of energy into a very confined space at the target site has enabled their use for multiple purposes, including cutting, sintering, welding, drilling, and surface functionalization across a wide range of materials.^1^ This versatility has led to the adoption of lasers in nearly all medical specialties, especially in dentistry.^2^ In dentistry, lasers have been employed for detecting caries, cutting soft and hard tissues, disinfecting root canals, and performing various surgical procedures like biopsies, gingivectomies, and frenectomies.^1,3^ During the 1980s and 1990s, Nd: YAG, Argon, CO_2_, and semiconductor diode lasers were mainly used for dental treatments.^4^ This was soon followed by the use of pulsed Er: YAG and Er, Cr: YSGG lasers to meet the surgical needs of clinical dentistry.^5^ Additionally, interest has grown in the use of ultrafast lasers—those with pulse widths from a few picoseconds to femtoseconds—in engineering and technology.^6^ The unique features of ultrafast laser processing, such as extremely high peak power and minimal heat diffusion to surrounding areas, have been extensively used in commercial, industrial, medical, and research applications, including micronano machining, 3D and volume processing, electronic chip fabrication, microfluidics, cutting and welding of various materials, clinical purposes, including diagnosis and treatment, and surface modification of many materials.^7,8^ Recently, the use of ultrafast lasers for the surface modification of various biomedical materials that require high energy and delicate processing has become increasingly prominent. Among these, ultrafast laser-based surface texturing of dental biomaterials is noteworthy. A brief description of various dental biomaterials is discussed in Section 1.1 below.

### 1.1 Dental Biomaterials

Dental biomaterials are artificial materials that are used to replace or restore damaged/diseased/lost parts of the dentition, as well as missing structures in the oral cavity, to reinstate function and improve aesthetic appearance.^9^ To be suitable for such applications, dental biomaterials must possess superior biocompatibility, mechanical, physical, and optical properties.^2^ Various materials used for this purpose can be conveniently grouped into four categories: metals and alloys, polymers, ceramics, and composites (Figure 1). Due to their inherent differences in buildup and structure, they exhibit distinct properties; hence, selecting a material with optimal properties that suit the given application is of utmost importance.

**Figure 1:**
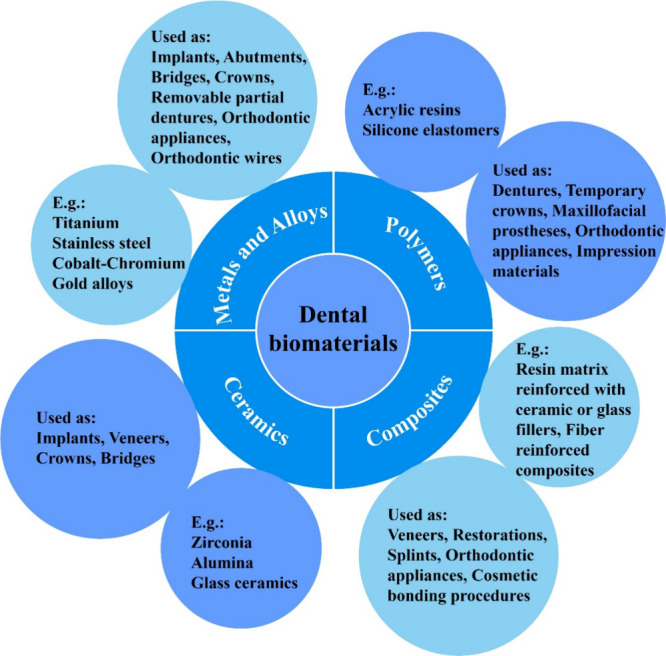
Classification of materials used in dentistry.

### 1.2 Dental Implants and Osseointegration with Bone

Conventionally, dental biomaterials have been used to replace the lost portion of the tooth structure, specifically, the coronal portion of the tooth structure. The groundbreaking research of Professor Per-Ingvar Brånemark regarding the osseointegration of titanium to living tissues led to the use of titanium-based implant materials to replace lost teeth.^10^ Commercially pure titanium (Cp-Ti) is the most widely used for making dental implants. Titanium as an implant material exhibits several advantages over other metallic materials, such as very low density, high strength, lower modulus compared to other materials, excellent corrosion resistance, and the ability to osseointegrate with the bone.^11^ Besides this, alloys of titanium (e.g., titanium-zirconia alloy), titanium with coatings, and zirconia are other materials used as dental implants.^9^ Osseointegration refers to the direct connection between the implant and the bone, with no intervening fibrous tissue.^12^ When the implant gets osseointegrated with the bone, a prosthetic component, like a crown or bridge, is placed over the implant using an abutment.^13^ The fact that implant osseointegration with the biological tissues occurs at the interface between the biological tissue and the implant surface, the surface characteristics of the implant, such as surface topography, chemistry, roughness, etc., play an important role in this process.^14^ Over the years, considerable research has optimized the surface characteristics of implants, leading to the development of commercial implants with a wide range of design features that favor initial stability, rapid osseointegration, and superior biocompatibility.

### 1.3 Microbial Adhesion on Dental Biomaterials

Despite their successful use as dental biomaterials, material-associated infections are still a major issue in the field of implant dentistry, affecting patient care.^15^ The major cause of these infections is microbial adhesion and the resulting biofilm formation.^16^ Oral cavities and biological fluids harbor a diverse population of commensal microorganisms, which under normal conditions maintain a balanced ecosystem. However, when present in elevated numbers or in environments conducive to the selective proliferation of specific microorganisms, these populations may aggregate and form structured biofilms. The development of such biofilms is facilitated by the production of an extracellular polymeric matrix that provides protection and promotes microbial persistence. These organized microbial communities can subsequently lead to localized or systemic infections, particularly when host defenses are compromised or when biofilm composition shifts toward pathogenic species.^17^ The biofilm formation can occur in five stages (Figure 2).^18,19^ The first stage is the initial attachment, in which the microorganism starts adhering to the surface and is unstable.^20^ In the second stage, the microbe forms a monolayer by interacting with the substrate surface using microbial adhesins. In this stage, the adhesion becomes irreversible. The third stage is the formation of multilayered microbial colonies, along with the discharge of extracellular polymeric substances (EPS), mainly polysaccharides and other macromolecules.^21^ In the fourth stage, the EPS develops into a 3D network within which the microbes grow, leading to the formation of a thicker biofilm (biofilm maturation).^22^ Lastly, some microbes disperse from the biofilm, going back to the independent planktonic lifestyle, and this cycle continues.^19^ As the extracellular polymer matrix, within which the mature biofilms get embedded, acts as a protective shield, it is difficult to eradicate the mature biofilms, as they are hard to treat with antibiotics. The intrinsic resistance to antimicrobial agents attained by microbial biofilms and the defense reactions of the patient’s immune system create difficulty in the treatment of microbial biofilms. Although the formation of a biofilm involves various stages, the first and foremost step is the ability of the microorganisms to establish an initial adhesion on the surface of the biomaterial. Hence, any method that can reduce the initial adhesion will help in reducing the incidence of infections due to biofilms on the dental biomaterials.^23^

**Figure 2:**
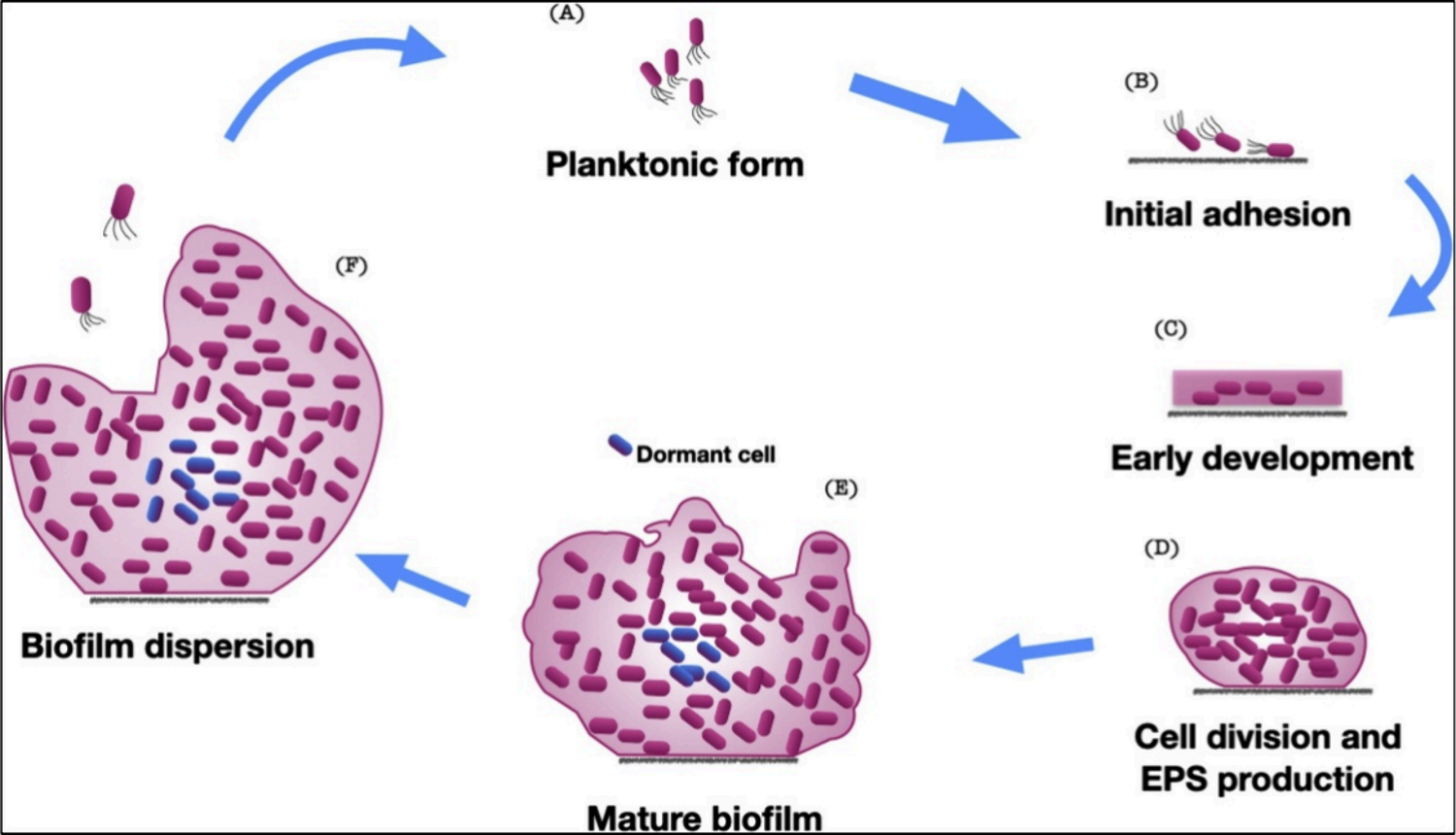
Different stages of microbial biofilm formation on biomaterials. Reprinted with permission from {Kreve and Reis, Japanese Dental Science Review} Copyright {2021} Elsevier.^19^

### 1.4 Role of Surface Properties in the Performance of Dental Biomaterials

Despite an implant being fully osseointegrated with the bone, it is likely that the abutment (the part through which the prosthesis or restoration is connected to the implant) can harbor microbes, leading to biofilm formation. The formation of microbial biofilms may lead to periimplantitis, a condition that affects both soft and hard tissues around the implant, leading to loosening and failure of the implant.^24^ To overcome this, the surface properties of the biomaterial have been modified suitably to reduce microbial infections and enhance cellular responses, thereby facilitating improved compatibility and bonding with surrounding biological tissues (both structural and functional).^25^ The surface topography as well as the roughness of the material were found to influence the osteoblast cell differentiation.^26^ Hence, surface topography is recognized as an important factor affecting the osseointegration.^27,28^

The initial adhesion of microorganisms onto any material is influenced by the species of microorganism, the size and shape of the microorganism, the type of substrate material, the physical and chemical features of the surface of the substrate, the environmental features, etc. Among these factors, the chemical and physical properties of the substrate surface are the most important properties that contribute to microbial adhesion. To be more specific, the chemical composition, surface charge, surface free energy, wettability, roughness, and surface topography of the substrate material play a vital role in microbial adhesion (Figure 3).^29^

**Figure 3:**
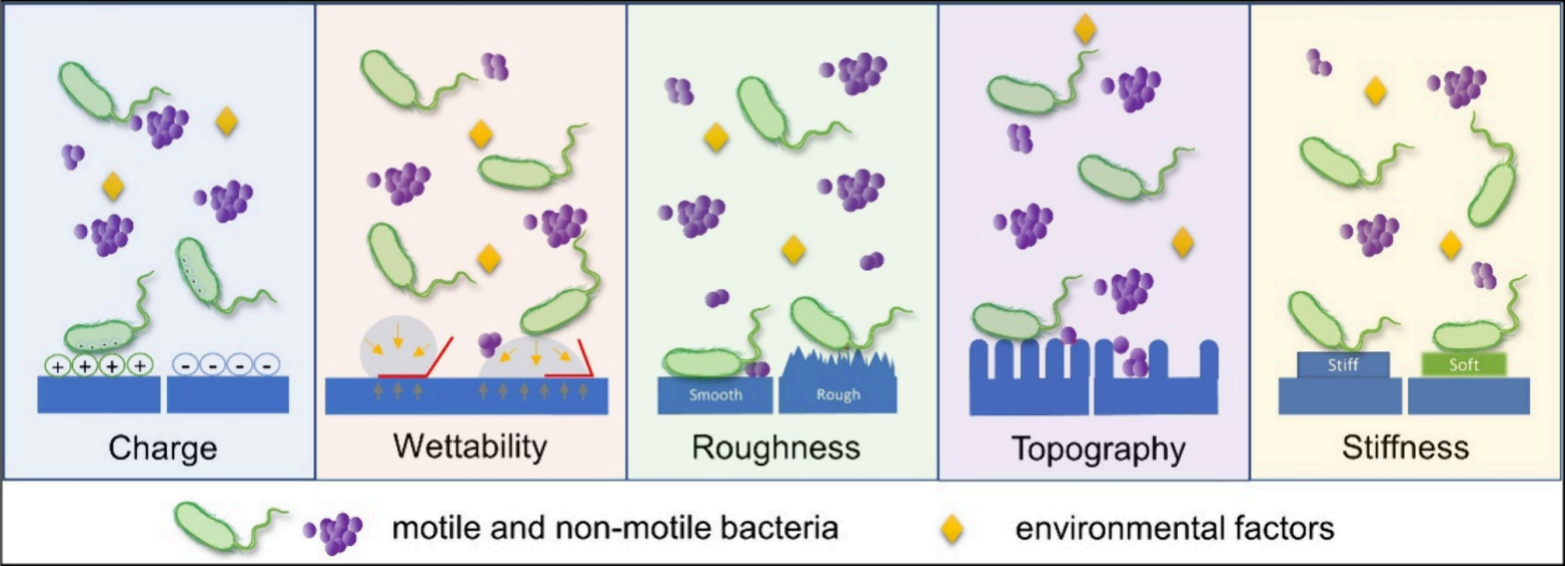
Major factors influencing microbial adhesion. Reprinted with permission from {Zheng et al., Frontiers in Bioengineering and Biotechnology} Copyright {2021} Frontiers.^29^

One important surface property that influences microbial adhesion is surface charge density. Due to the presence of carboxyl, amino, and phosphate groups on their cell wall surfaces, it is considered that bacteria have a net negative charge. Hence, bacteria tend to adhere to positively charged surfaces.^30^ The surface free energy and, thereby, the wettability of a surface are also factors that affect microbial adhesion. In the case of liquids with low surface tension and materials with high surface energy, the surface wettability will be high. In other words, the liquid tends to maximize its area of contact with the material surface and wets the surface. Antiwetting surfaces can be fabricated by minimizing the surface energy. However, several studies have demonstrated that attaining extreme water contact angles (superhydrophobic/superhydrophilic) could resist microbes.^29^ The effect of the roughness of the material surface on microbial adhesion has been extensively studied. Smooth surfaces showed increased microbial adhesion. In contrast, nanoscale roughness on the material surface reduces the surface area in contact with the bacterial cells, leading to decreased bacterial adhesion.^31^ Studies have also shown that materials with surface features having dimensions greater than the size of the microorganism cause increased adhesion as such surfaces provide a large space for their cells to attach. Hence, substrate materials with surface features smaller than the bacterial size may lead to reduced bacterial adhesion.^32^ However, the prime factors influencing microbial adhesion, as considered by the majority of the research, are material surface topography, roughness, and wettability. So, to fabricate the antibacterial prosthetic material, the surface should possess characteristic features that inhibit the adhesion of microorganisms. This put forward the concept of surface modification of the substrate material to enhance the osseointegration of implant materials to the bone and to resist microbial adhesion.

### 1.5 Various Surface Modification Methods

Even though there are many different ways of surface modification, they are broadly classified into two methods: additive and subtractive.^33^ The additive method involves adding some other material or chemical to the prosthetic material, either by coating or by impregnating.^34^ However, these coatings may not be long-lasting, and the osseointegration potential and biocompatibility will depend on the chemical constituents of the coatings and their stability. Meanwhile, the subtractive method involves either the removal of material from the surface or the making of plastic deformation on the material surface to create roughness on the surface.^34^ The most commonly used subtractive methods in implant dentistry are large-grit sand or ceramic particle blasting (SLA), acid etching, etc.^35^ Grit blasting involves blasting sand-like particles of hydroxyapatite, alumina, or titanium dioxide (TiO_2_) under high pressure onto the implant surface.^34^ The surface topography of a grit-blasted implant material varies depending on the size of the particles used, the pressure applied during grit blasting, the duration of grit blasting, etc. Acid etching involves the use of strong acids like hydrofluoric (HF), nitric (HNO_3_), and sulfuric (H_2_SO_4_), or their combination, to induce roughness on the material surface.^36^ During such treatments, localized removal of the material leads to the creation of a variety of surface topographies, which are dependent on the combination of acids used, their strength, and the duration of the exposure. The aforementioned methods have been widely used either alone or in combination to enhance the osseointegration potential of dental implants.^37,38^ Although they have largely been successful, there are two potential drawbacks with these methods: first, it is difficult to have different surface topographies on different parts of the implants, and second, the possibility of remnants of the surface modification agents being on the implant surface.^9^ In this regard, laser-induced surface modification, which is also a subtractive method of surface modification, is of great importance. Laser-assisted surface treatment, which is a noncontact treatment method, affects only the surface layer of the specimen without altering its surface composition, leaving no contamination, nor impacting the bulk properties of the specimen.

Recently, the clinical demand in dentistry has been for developing multifunctional biomaterials with surfaces that exhibit superior biocompatibility, environment-responsive behavior, bioactivity, antimicrobial activity, etc.^39^ The success or failure of the implant in a biological system is greatly influenced by the interactions at the interface between the implant and the biological tissue. Concerning this, the surface aspects of the biomaterials, such as accelerating the osseointegration, exhibiting antimicrobial activity, and responding to biological stimuli, become even more important. Hence, various techniques for modifying the substrate surface have been developed to foster bioacceptance and also to reduce microbial adhesion. Although the actual biomaterial surface is smooth, they are intentionally machined to induce threadlike structures and macrosurface features to significantly increase the area of contact and to minimize shear forces at the interface between the implant and the bone. Over the years, additional surface modification in the form of micro- or nanolevel has been widely investigated to achieve better osseointegration by modifying the surface characteristics like surface energy, wettability, and roughness or even by incorporating ions, nanoparticles, or active molecules on the material surface. In contrast to the conventional grit blasting and acid etching methods, lasers, in recent times, have been widely used as potential tools for modifying the surface of various biomaterials. Figure 4 shows the statistics of publications on laser-assisted surface modification of biomaterials based on Scopus search, using the keywords “laser”, “surface modification”, “dental biomaterials”, “titanium”, “PMMA”, and “zirconia”, as of 28th April 2025. These studies include various laser-based surface modification techniques such as direct laser writing, laser-based surface melting, direct laser interference patterning, laser-based chemical modifications on biomaterial surfaces, etc., over the past 25 years. The data clearly show that the research focus in the field of laser-assisted surface modifications of biomaterials is growing rapidly, especially after 2010, indicating increased advances in this field. Among these studies, more attention has been paid to the analysis of the physical and mechanical properties of laser-textured biomaterials. The research focus on the osseointegration of biomaterials with bone is also rapidly evolving, while antimicrobial research has received slightly less focus, representing an important area for future exploration.

**Figure 4:**
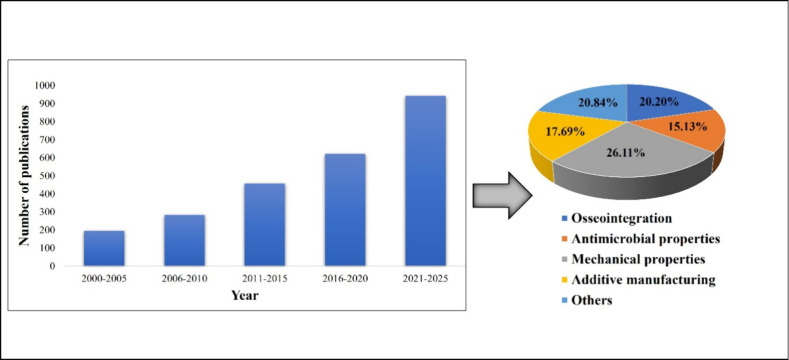
Statistics of publications on laser-assisted surface modification of biomaterials based on Scopus data as of 28th April 28th, 2025.

Considering the increased utilization of lasers in modifying implant biomaterial surfaces, this review aims to provide readers with a comprehensive understanding of the role of lasers in the surface modification of dental biomaterials, enhancing osseointegration and minimizing microbial adherence. This review further seeks to offer thorough knowledge of controlled laser ablation, the influence of various laser parameters on the material surface properties, and how these surface characteristics impact the overall performance of various dental biomaterials. The present review is organized as follows: First, the methodology for selecting relevant studies on surface modifications of dental implant materials including the search engines and specific keywords and the inclusion criteria are outlined in Section 2. Subsequently, key findings from the articles on the influence of various laser parameters on the surface features of various materials and subsequent changes in the properties such as roughness and wettability were discussed in Section 3.

## 2 Laser Processing: A Versatile Approach to Tailor the Surface Topography of Materials

A literature search has been conducted in databases such as “Google Scholar”, “Scopus”, and “PubMed” covering the years from 2000 to 2025. The terms used for searching included “laser”, “surface modification”, “dental biomaterials”, “ultrafast”, “laser texturing”, “osseointegration”, “microbial”, “adhesion”, “cell adhesion”, “wettability”, “roughness”, “patterning”, “titanium”, “PMMA”, “zirconia” “PEEK”, “implant”, “ceramics”, and “microtexturing”. Manuscripts of all types are included for review and are in English. After the compilation of the results from various databases, 268 manuscripts were found to meet the criteria, which included studies on laser-assisted surface modification of various biomaterials, laser-based chemical modification, and additive manufacturing studies, and studies on the effect of laser treatment on biological properties like cell differentiation and growth, microbial adhesion, physical and mechanical properties like hardness, flexural strength, bonding efficiency, and wear and corrosion resistance. Among these, only those that are relevant for the laser-based surface modification of dental biomaterials were selected, which were 132. For each of these manuscripts, the abstracts were initially read to understand the scope of work. Only those studies that focused on the implementation of direct laser texturing, more specifically ultrafast lasers, for surface modification of dental biomaterials, which studied their effect on biological responses, such as osseointegration and microbial adhesion, were included. Those studies that gave importance to other laser-based surface modification techniques like direct laser interference patterning, selective laser melting, etc., and those studies that analyzed the effect of laser texturing on material properties like bond strength, hardness, porosity, and wear resistance were excluded. Finally, 123 research articles were included for analysis in this review, out of which 94 were exclusively focused on the use of lasers for surface modification of dental implant surfaces.

Based on the literature search for this review, numerous in vitro and in vivo studies have investigated the effect of laser-assisted surface texturing on various surface features, including wettability, surface topography, and roughness, and the effect of such modification on the biological response, such as differentiation, growth, and proliferation of cells and microbial adhesion and subsequent formation of biofilms. The impact of laser-based surface modifications on the physical and mechanical properties of biomaterials, such as hardness, strength, bonding efficiency, corrosion resistance, porosity, wear resistance, etc., has also been widely explored. To be more specific, 23% of the research articles considered in this review studied the cell viability and adhesion behavior (osseointegration) of various dental biomaterials, and 30% of the articles dealt with the study of antimicrobial properties of various dental biomaterials. 16% of the articles studied both the antimicrobial as well as the cell adhesion behavior of dental biomaterials. The remaining 31% of the articles dealt with other studies, like analyzing topography, roughness, wettability, etc., of laser-patterned material surfaces. Moreover, much of the research considered in this review adopted ultrafast lasers for surface modification of the respective material. When considering the substrate materials used for laser-based surface modification, 45% of the studies were on titanium. This indicates that most laser-based surface modifications have predominantly been applied to implants, especially those made of titanium and its alloys, to assess their potential in enhancing osseointegration. In addition to dental implants, laser-surface texturing has also been employed on ceramic restorations to enhance their bonding efficiency to teeth. Research on laser-based surface modification of polymer materials like PMMA, PEEK, etc., is comparatively less.

### 2.1 Laser-Assisted Surface Modification

Recent developments in the field of laser technology have paved the way for its widespread application across various fields. Among these applications, utilizing lasers for material surface modification to improve its characteristics, such as biocompatibility, roughness, wetting, and mechanical properties, corrosion resistance, etc., is also gaining much importance in the current scenario.^40–42^ During laser machining, the laser beam is focused on the workpiece, which can be metal, ceramic, or plastic. If the laser beam has energy higher than the binding energy of the electrons of the atoms of the workpiece, then laser ablation occurs. In this process, the material removal rate is influenced by the type of workpiece as well as the laser system parameters like wavelength, pulse duration, pulse fluence, etc. Based on the mode of operation, lasers can be categorized into continuous wave (CW) lasers (continuously delivering photons) and pulsed lasers (delivering photons in short bursts or pulses). Contrary to CW lasers, which are characterized by constant output power, pulsed lasers are capable of delivering very high peak powers of the order of megawatts, even for moderate energies.^43^ This enables the rapid discharge of stored energy onto the target material. Hence, pulsed lasers with intense peak power are effectively utilized to pattern the surface of different materials via laser ablation. Moreover, repeatable as well as more precise and accurate small holes/patterns/craters can be made using laser micromachining, which is a noncontact micromachining technique. Some other advantages of laser micromachining over conventional micromachining techniques are a very minimal to zero heat-affected zone, obtaining burr-free cuts, spatial selectivity, etc.

There are several laser surface engineering technologies for fabricating specific structures on a material. They include direct laser interference patterning (DLIP) or laser interference lithography (LIL), selective laser melting (SLM), direct laser ablation, etc. (Scheme 1 and Table 1).^44^

**Scheme 1:**
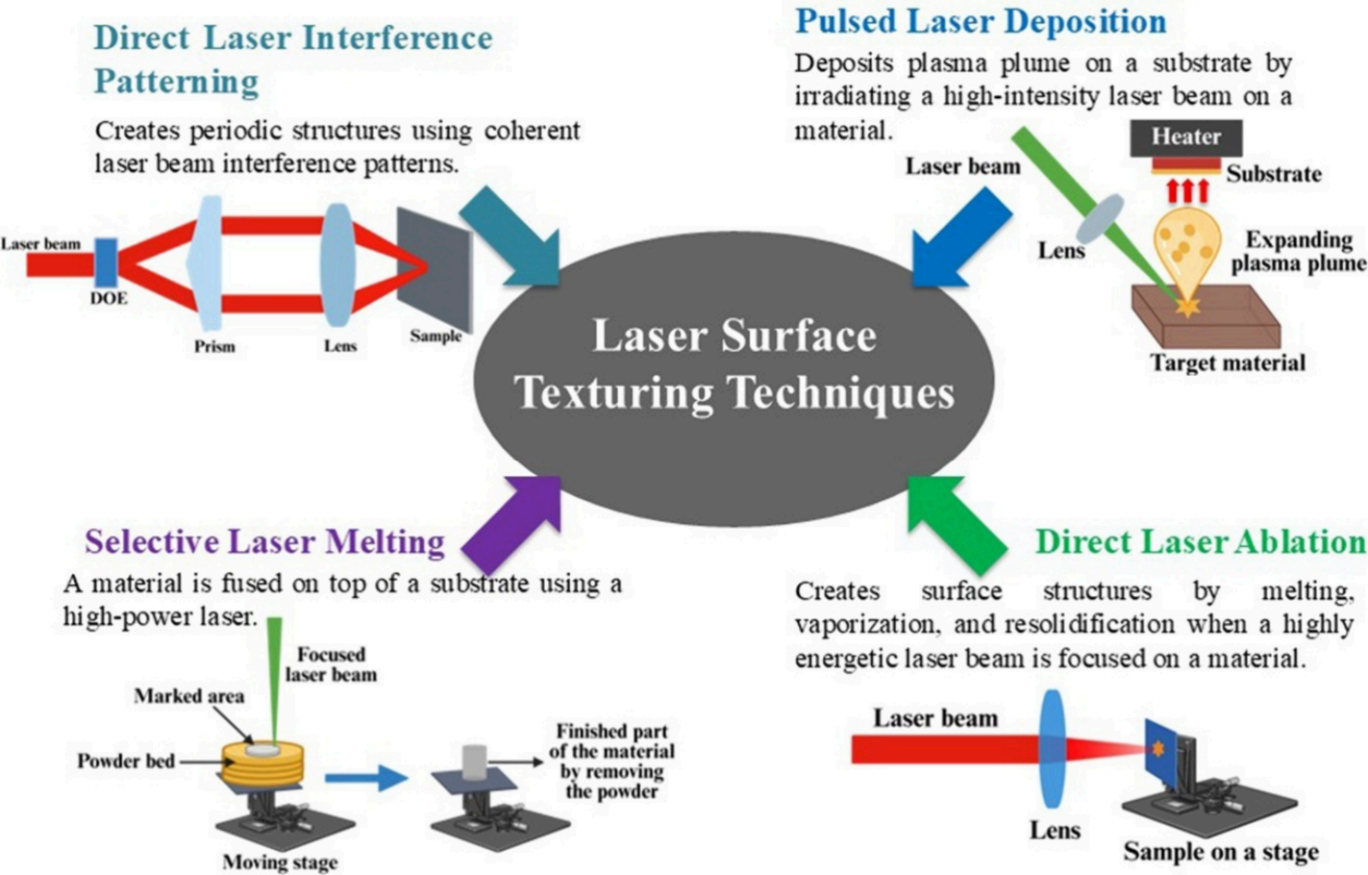
Various Laser-Based Surface Texturing Techniques^44–47^

**Table 1:** Different Laser Processing Techniques for Creating Specific Surface Structures on a Material

method	brief description	advantages	disadvantages	applicable material types	clinical application scenarios
Direct laser interference patterning (DLIP)^45,48,49^	• Makes use of the concept of coherent laser beam interference: a coherent laser beam is split into several beams that overlap on the substrate surface, creating interference patterns (periodic structures) whose size is determined by the laser wavelength and the angle between the interfering beams.	• Commonly used to create complex three-dimensional surface structures.	• Limitations in the variation of laser energy distribution within the interference patterns make the fabrication of high aspect ratio surface structures quite challenging.	• Metals and alloys	• Widely used for treating various biomaterials to enhance their biocompatibility, tribological properties, antibacterial properties, etc.
		• Suitable for processing large areas, as the processing area depends on the area of the laser-interference field.		• Polymers	
Pulsed laser deposition (PLD)^47,51^	• Involves the deposition of a plasma plume on a substrate, in which the plume is produced by irradiating a material with a high-intensity laser beam.	• Deposition efficiency is high.	• Difficult to maintain uniform deposition on large areas.	• Metals and alloys	• To deposit coatings (such as calcium hydroxyapatite (HA)) on orthopedic and dental implants.
		• Adheres strongly to the specimen.		• Metal oxides	
				• Ceramics	
Selective laser melting (SLM)^46,52^	• An additive manufacturing technique that makes use of a high-power laser to selectively melt and fuse a material on top of a base material.	• Capable of achieving a 3D geometry by melting the consecutive layers of a material.	• There are chances of forming cracks and pores on the surface structures, which may potentially compromise the substrate’s mechanical properties.	• Metals (mostly used)	• To prepare metal frameworks for dental prostheses.
				• Ceramics and composites	
Direct laser ablation^45,53^	• A highly energetic laser beam is focused to ablate the material of interest to create micro/nanostructures on the surface by rapid melting, vaporization, fragmentation, resolidification, etc., of the material.	• Accurate material removal at the micron/submicron level is possible.	• Precision in the texturing is often limited by the type of laser, the spot size of the beam, and the pulse energy.	• Metals and alloys	• Extensively used in the field of tissue engineering and numerous other medical-related industries to improve the biocompatibility, antimicrobial properties, differentiation, and proliferation of cells.
		• Mostly employs pulse-to-pulse strategies.		• Ceramics	
				• Polymers	

Among the above-mentioned laser processing techniques, this review majorly focuses on direct laser ablation of dental biomaterials, a simple and effective method of creating surface structures.

### 2.2 Mechanism of Direct Laser Surface Texturing

The use of lasers for precise and accurate material processing is possible only with deep insight into the interaction of laser radiation with matter. The actual physics of laser-matter interactions, when a highly intense laser beam is incident on a material, can be described as follows. Consider a photon incident on a neutral atom. If the energy of the photon is high enough to break the binding energy of the electron in the atom, then the electron becomes free and the atom gets ionized. But for some atoms, a single photon energy may not be sufficient to get ionized. In such cases (at high optical intensities), the atoms undergo two-photon absorption (a process in which two photons are simultaneously absorbed, exciting the atom to a higher energy state). Some atoms may also absorb multiple photons via virtual states to get ionized and release free electrons, which is termed multiphoton absorption. In such cases, when a laser beam having high intensity interacts with a material, some of the bound electrons undergo multiphoton absorption and become free electrons.^54^ When such a free electron comes to the vicinity of a neutral atom in the presence of a photon (a three-body system), the atom gets ionized—the case of inverse bremsstrahlung. These free electrons again cause the ionization of other atoms, thereby producing more free electrons. This results in a state of electron avalanche (Figure 5).^54^ When a critical free electron density is achieved, complete breakdown of the material occurs and the material gets ablated.

**Figure 5:**
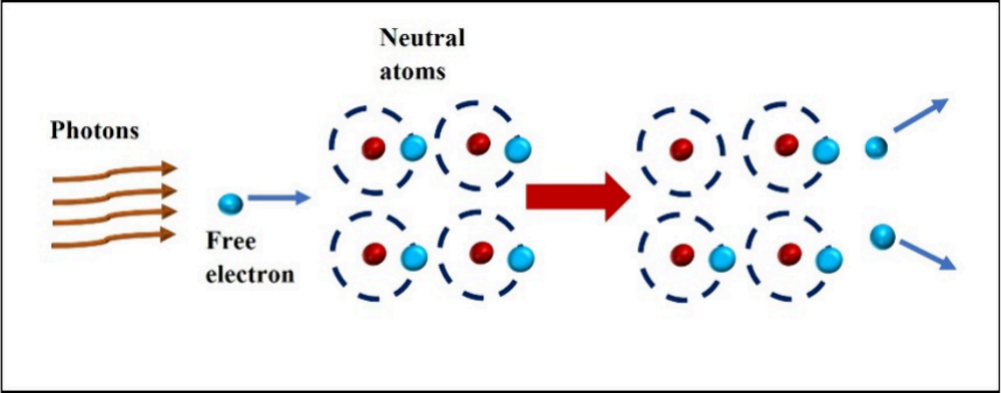
Various processes associated with avalanche ionization.

In simpler words, a laser beam focused on a material surface results in energy absorption. However, the energy absorption mechanism is highly influenced by the wavelength, repetition rate, pulse width, etc. Depending on the laser parameters, either controllable surface modification or direct material removal is possible. The energy absorption mechanism also depends on the type of material on which the laser beam is irradiated. For example, the interaction of laser energy with metals and polymers is much too different. In the case of metals, the conduction band electrons directly absorb photons and transit to higher energy states. However, for materials like ceramics or polymers, the laser energy absorption occurs only at high intensities, as they have a wide band gap.^53^ To be more specific, as the valence electrons of metals are loosely bound, less energy is required for the excitation and ionization of electrons compared to ceramics and polymers, whose backbone elements (such as carbon, silicon, etc.) possess more tightly bound valence electrons. This difference in binding energy suggests why pulsed lasers with high intensities are highly preferred for the laser surface texturing of ceramics and polymers, compared to metals.^1^ In addition to this, ceramic materials are prone to having microcracks due to the laser-induced thermal stress, which can be avoided by minimizing the intensity of the plasma plume during the laser-material interaction. In the case of polymers, even though energy absorption occurs at high laser intensities, the chances of thermal degradation of the material along with photoablation are quite high.^55^ The thermal damage can be reduced to an extent by utilizing ultrafast lasers for material processing, which transfers energy to the material before thermal diffusion.^56^ Hence, there is no doubt that the excitation dynamics and their influence on material surface quality are highly complex as both depend intricately on the specific material type and the applied laser parameters.

### 2.3 Ultrafast Lasers for Surface Modification

Over the past few years, the use of ultrafast lasers for surface texturing of dental biomaterials has become a widely investigated area. For pulsed lasers, the mechanism by which a laser pulse interacts with a substrate material is determined by its pulse width (pulse duration). Depending on the pulse duration, laser pulses can be classified as long pulses (typically ranging from tens of nanoseconds to hundreds of microseconds) and ultrashort pulses or ultrafast pulses (ranging from tens of picoseconds to femtoseconds). Several studies have reported how laser pulses with different pulse durations interact with materials, showing that nanosecond laser ablation generated a thicker recast layer than ultrafast laser ablation.^57,58^

The interaction of nanosecond, picosecond, and femtosecond laser pulses with materials is briefly described below, and a comparison between them is shown in Table 2. In the case of nanosecond pulses (long pulses), the time for the heat energy to traverse the material is longer. So, the laser irradiation absorbed by the material heats its surface, resulting in the formation of a large layer of molten material. The vaporization process that occurs further causes the liquid (molten material) to be expelled. Thus, for nanosecond laser pulses, material removal occurs in both the vapor and liquid phases. This makes the laser processing less precise. Another problem seen with nanosecond laser processing is plasma shielding. The particles generated by laser ablation interact with a portion of the irradiated laser beam, reducing the amount of energy that reaches the surface and, consequently, lowering the ablation rate.

**Table 2:** Table Showing the Comparison between Nanosecond, Picosecond, and Femtosecond Lasers^59,61^

characteristics	nanosecond laser	picosecond laser	femtosecond laser
Pulse duration	10^–9^ s	10^–12^ s	10^–15^ s
Mechanism of ablation	Thermal ablation of material through melting and vaporization	Ablation (nonthermal) through melt formation within the material due to heat conduction by electrons	Ablation through energy absorption by free electrons causes rapid heating in a picosecond time regime, and hence, the melt phase is absent
Thermal effect	High thermal effect leading to more heat-affected zones	Low thermal effect, thereby less damage to the surroundings	Minimum to zero thermal effect leading to ablation within a well-defined area
Plasma shielding	High plasma shielding	Minimum plasma shielding	Negligible plasma shielding

For picosecond laser pulses (short pulses compared to nanosecond pulses), the laser irradiation leads to heat conduction by the electrons in the material, and thus, a melted region is formed inside the target material. Along with this, a transition from solid to vapor occurs directly at the material surface. However, inside the material, the molten substance (liquid) minimizes the precision of laser texturing.

For femtosecond laser pulses (ultrashort pulses), the time scale for ablation is very short. The femtosecond laser pulse irradiation on a material directly transits the material from the solid state to the plasma state. Upon laser irradiation, free electrons absorb the incident energy and transfer it to the lattice, causing rapid heating on a picosecond time scale. This results in vaporization and plasma generation, followed by a sudden expansion in a vacuum. The conduction of heat into the material is negligible during this process. So, in femtosecond laser processing, because of the immediate generation of vapor and plasma states, thermal dissipation becomes negligible, and hence, the molten phase is absent.^59^ Plasma shielding is also negligible in the case of femtosecond lasers. As a result, this makes it possible to ablate the material with very minimal laser fluence. However, at high laser intensities, some nonlinear absorption processes dominate over the above-mentioned nonthermal processes.^60^ In other words, the high intensity of lasers induces nonlinear absorption processes such as multiphoton absorption, avalanche ionization, etc., resulting in the generation of free electrons with sufficient density to trigger the ablation, regardless of the type of sample material.

As seen in Figure 6, for long-pulsed laser ablation, the Heat-Affected Zone (HAZ) is maximum, and the laser surface treatment becomes less precise due to the presence of microcracks, the recast layer, and the resolidified layer. For ultrafast laser ablation, the formation of surface debris, microcracks, as well as heat-affected zones is very minimal, and the fabricated grooves have clear edges.^62^ They are capable of ablating material from the surface quickly and cleanly without affecting the surrounding regions. From the aforementioned discussion, it is clear that ultrafast lasers can create nano- or micropatterning structures on the surfaces of various materials without significantly affecting the surrounding areas. Hence, they have been extensively employed for surface modifications of various biomaterials to enhance osseointegration and antimicrobial properties. A detailed discussion on this is presented in Section 3 below.

**Figure 6:**
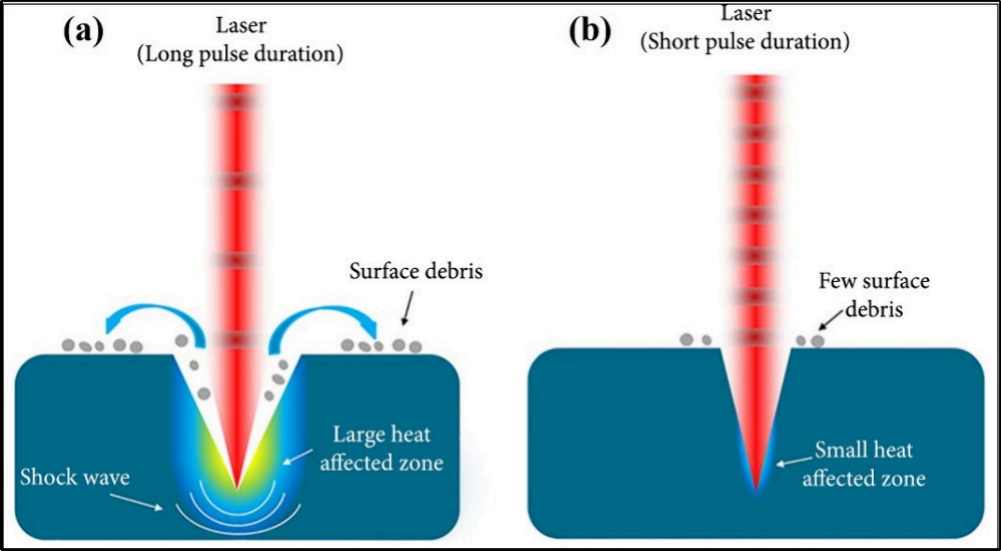
A comparison between (a) nanosecond (long pulse duration) and (b) femtosecond (short pulse duration) laser-treated material surfaces. Reprinted with permission from {Lin and Hong, Ultrafast Science} Copyright {2021} Science Partner Journals.^61^

## 3 Influence of Various Laser Patterning Parameters on the Material Surface Properties

### 3.1 Basic Mechanism of Direct Laser Texturing

Laser-assisted surface modification systems usually consist of a high-energy laser beam, which is focused on the substrate material of interest placed on a translation stage using suitable optics (Figure 7). The surface characteristics of the laser-processed substrate highly depend on the mode of operation of the laser, more specifically, the laser parameters. Once a laser-based surface modification system is developed, various laser parameters, such as the type of laser, wavelength, energy, pulse width, repetition rate, etc., have to be optimized for creating the desired textures on the sample material surface. The laser wavelength will be chosen based on the optical characteristics of the sample material of interest. For example, the optical reflectivity of metals is very high in the near-infrared region compared to the UV (shorter) wavelength region. But at shorter wavelengths, the maximum laser power available tends to decrease, and hence, lasers with visible wavelengths are preferred for the ablation of metals.^55^ The value of the laser fluence also depends on the substrate material chosen for the surface modification. Compared to polymer materials, the laser fluence for surface patterning on metals is generally high, as their ablation threshold is high. As a quantitative explanation, for a solid material irradiated with a 100 fs laser pulse, the excitation occurs in the femtosecond range and the process of melting occurs roughly in the picosecond regime. The ablation and, thereby, material removal last up to the nanosecond time regime.^63^ The range of intensities for this to happen spans from approximately 10^10^ W cm^–2^ to beyond 10^14^ W cm^–2^ in the case of metals. For ceramics and polymers, this range is even higher.^53^ The range of energy required for surface ablation depends mainly on the type of material and its chemical makeup and structure, as outlined in Section 2.2. The amount of laser energy plays an important role in determining the shape, width, and depth of the microstructure created.^64^ In the case of repetition rate, a high value results in a large number of laser-ablated craters per unit area. By variation of these laser parameters, craters with different dimensions can be created on the substrate surface. Similarly, by varying the speed of translation of the motorized stage and its direction of movement, the distance between the craters, i.e., hatch spacing (distance between two consecutive laser spots) and, thereby, pulse overlapping can be adjusted for fabricating different structures on the substrate surface. Each of the patterning parameters influences the interaction between the laser beam and the surface of the materials, thus influencing the rate at which the material is ablated. The rate of material ablation also depends on the laser processing atmosphere. For example, the ablation efficiency of a material is high in a vacuum compared to ambient air.^65^ Thus, the influence of laser patterning parameters on the spacing density, roughness, and wettability will be studied by analyzing the surface patterns created. Among these patterns, optimized patterns will be selected based on the surface characteristics for further evaluation. For this purpose, the laser-textured material was subjected to various surface analysis techniques. The various techniques commonly used to analyze the morphology of the laser-textured surface include Scanning Electron Microscopy (SEM), Optical Microscopy, Atomic Force Microscopy (AFM), field electron-scanning electron microscopy (FE-SEM), etc. Wettability studies are usually carried out by analyzing the water contact angle measurements, mostly done by using a contact angle goniometer. Surface roughness is analyzed using methods such as Scanning Electron Microscopy (SEM), Confocal Laser Scanning Microscopy (CLSM), profilometry, etc., and techniques that use scanning probes, such as Atomic Force Microscopy (AFM).^66,67^ In addition to this, researchers also evaluate the changes in surface chemistry after laser-texturing through techniques such as Energy Dispersive X-ray Spectroscopy, X-ray Diffraction, X-ray Photoelectron Spectroscopy, Raman Spectroscopy, etc. To be more specific, the laser processing parameters have to be adjusted to create specific surface structures such that these structures would serve particular functions, such as enhancing cell adhesion and proliferation or inducing antibacterial properties (Scheme 2). A parametric study is performed to evaluate the best processability ranges, thereby choosing the best set of parameters that creates surface features that will enhance the surface roughness, for controlling the wettability. The control of surface wettability is essential for either inhibiting microbial adhesion or enhancing osseointegration. Hydrophilic surfaces are the most preferred for the enhancement of adhesion, whereas hydrophobic surfaces are used for the generation of self-cleaning surfaces and reducing adhesion.

**Figure 7:**
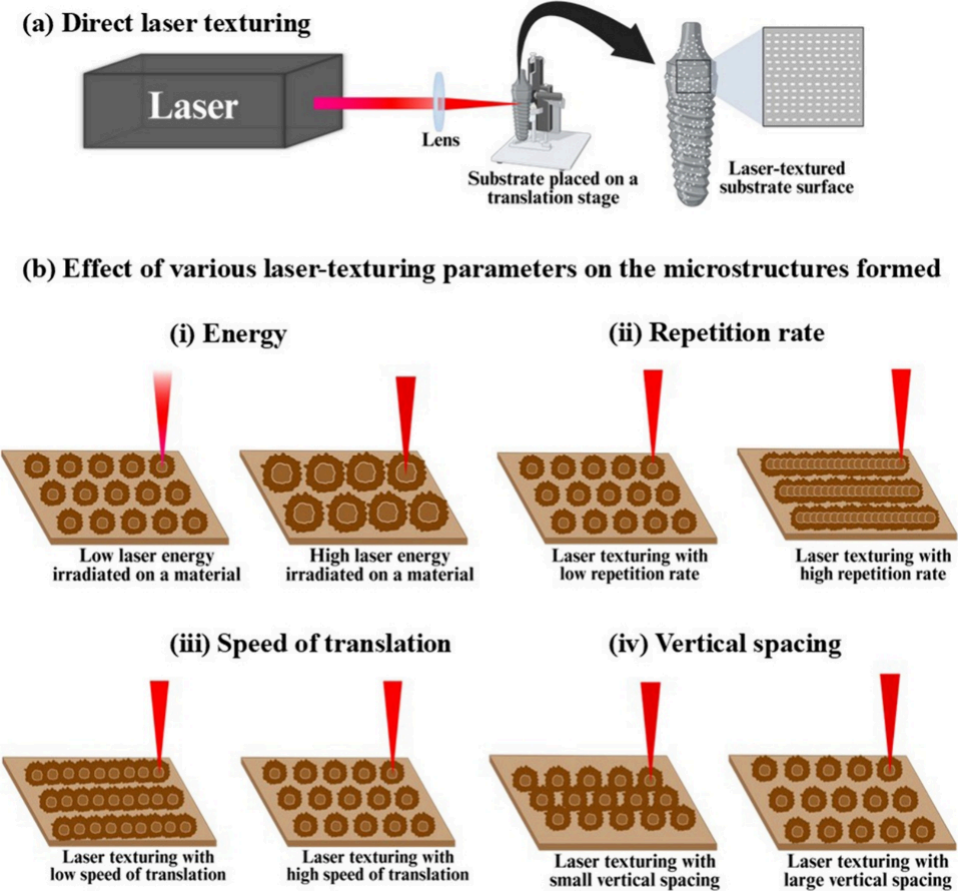
(a) Schematic representation of direct laser surface texturing and (b) effect of various laser-texturing parameters on the microstructures formed.

**Scheme 2:**
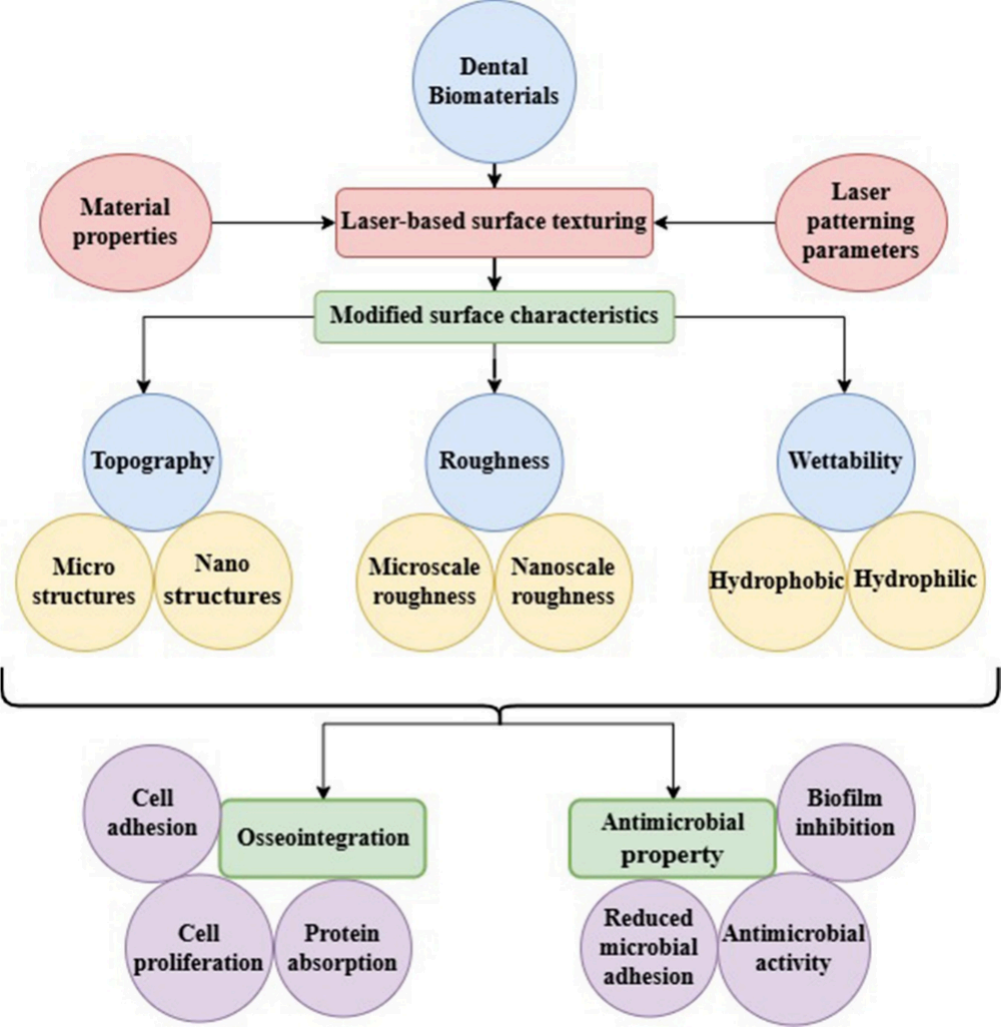
A Conceptual Diagram Showing the Relation between Laser-Assisted Surface Texturing of Dental Biomaterials, the Resulting Surface Features, and Biological Responses

Optimization of the best combination of laser patterning parameters is usually done experimentally. In addition to this, methods of design of experiments (DoE), such as the Taguchi method, neural network, etc., are also adopted to support the experimentally selected combinations of laser patterning parameters. The initial step of the Taguchi method involves the selection of the processing parameters based on experimental analysis and then the choice of a proper orthogonal array to carry out the experiments. The experimental results are then analyzed using analytical methods, like the Signal to Noise ratio (S/N) ratio, which represents the ratio of meaningful value for the output response and the power of background noise (unwanted information), and statistical Analysis of Variance (ANOVA) by design-expert software. The analysis ultimately helps determine the best suitable combination of process parameters.^68,69^ The ANOVA method is conducted to study the impact of individual processing parameters on the observed output and to find which parameters have a significant effect on the laser-texturing.^70^ Evaluating the applicability of artificial neural networks to establish a nonlinear relation between laser-ablated microstructures and the laser patterning parameters has also become a recent matter of interest.^71^ Such methods can be powerful tools for the statistical design of experimental parameters, considering multiple factors so that they reduce the test time. The following section deals with the effect of laser-texturing on various surface parameters.

### 3.2 Topography

Texturing of a material surface using lasers can generate diverse surface structures that are micrometer or nanosized. Laser’s ability to create regular and periodic surface structures is being widely used for various surface engineering applications. Surface features commonly generated by laser-based techniques include Laser-Induced Periodic Surface Structures (LIPSS), microchannels or microgrooves, microholes, micropillars, porous structures, and hierarchical structures (Figure 8). A particular combination of laser parameters will produce a distinct surface structure. Uhlmann et al.,^72^ in their study, have produced various surface structures such as microchannels, microcavities, and LIPSS on titanium alloy through laser texturing with different combinations of scanning speed and laser fluence.

**Figure 8:**
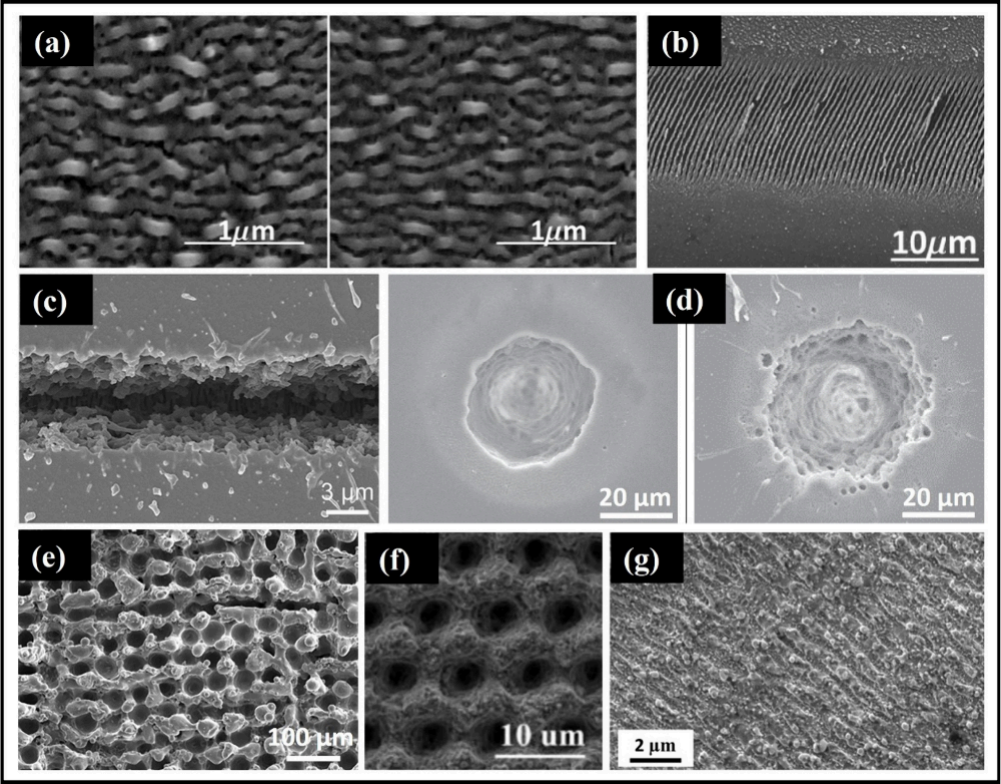
(a and b) SEM images of typical High Spatial Frequency LIPSS and Low Spatial Frequency LIPSS on the titanium film surface. Reprinted with permission from {Nathala et al., Optics Express} Copyright {2015} Optica Publishing Group.^73^ (c) SEM images of a microchannel (microgroove) prepared on the PMMA surface. Reprinted with permission from {Ouyang et al., Optics & Laser Technology} Copyright {2022} Elsevier.^74^ (d) SEM image of a microhole on the PMMA surface. Reprinted with permission from {Sunderlal Singh and Samuel, Optics & Laser Technology} Copyright {2024} Elsevier.^75^ (e) SEM image of micropillars on the titanium surface. Reprinted with permission from {Patel et al., Surface and Coatings Technology} Copyright {2018} Elsevier.^76^ (f) SEM image of the porous structures on the silicon surface. Reprinted with permission from {Zhao et al., Applied Surface Science} Copyright {2015} Elsevier.^77^ (g) SEM images of a hierarchical surface texture on titanium. Reprinted with permission from {Gupta et al., Langmuir} Copyright {2024} ACS Publications.^78^

Laser-induced periodic surface structures (LIPSS) are nanoscale-sized surface structures having periodic ripple-like topography.^79^ LIPSS-based surface patterning has been recently investigated for several applications, such as enhancing the adhesion capability and controlling the wettability of different materials, etc. Based on the periodicity, LIPSS are classified into two categories: Low Spatial Frequency LIPSS (LSFL), having a period between λ and λ/2, where λ is the wavelength of the laser beam, and High Spatial Frequency LIPSS (HSFL), having a period below λ/2.^80^ Femtosecond laser-based surface texturing on dental implants made of pure titanium has been reported to generate LSFL on the crest portion of the implant and HSFL on the highly inclined flank portion of the implant by adopting appropriate patterning parameters, and the surface structures were found to increase the attachment and proliferation of the osteogenic cells.^80^ In addition, the potential of LIPSS with extremely uniform nanostructures, called Highly Regular LIPSS, fabricated using a femtosecond laser in the enhancement of osteoblast cell adhesion, has been evaluated and was found to be effective.^81^

Other typical laser-fabricated surface structures are microchannels and microgrooves, almost similar structures, which are widely utilized, generally in microfluidic applications. Several studies have fabricated such structures in dental biomaterials for various applications. Regular arrays of microgrooves and ridges on dental zirconia fabricated using a femtosecond laser have been reported to improve the early stage differentiation of preosteoblast cells. Sunderlal Singh and Samuel^75^ analyzed the variation of the dimensions of a femtosecond laser-ablated microchannel on PMMA with various patterning parameters. Their study reported that both the width and depth of the microchannels increased with an increase in the laser energy and the number of laser pulses. Moreover, an increase in the scanning speed resulted in a decrease in the channel width. Microchannels were created on PMMA with a width less than the beam waist by choosing an energy value comparable to the ablation threshold of the material and an optimum value of the scanning speed. This shows the efficiency of an ultrafast laser system in creating structures less than the focal spot without the use of lenses with shorter focal lengths to reduce the focal spot size.

Direct laser ablation also produces microhole structures, the dimensions and morphology of which depend upon the laser parameters such as laser fluence, pulse duration, and number of pulses.^82^ The microhole diameter and depth increase with the increase in energy density and number of laser pulses and may also lead to the formation of increased melts and debris, the resolidification of which produces bead-like or wave-like structures within the microhole.^83^

Micropillar or microcolumn-like and microporous structures have been found advantageous for wettability applications. These structures increase the roughness and lead to the formation of a solid–air–liquid interface, increasing the hydrophobicity of the material surface. Surface texturing of titanium alloy using a femtosecond laser in a study has been observed to produce two different surface morphologies: microchannel-like structures, made at low scanning speed, and microporous structures with a lot of protrusions, made at high scanning speed.^84^ Surfaces with microporous structures had a hydrophobic nature, which exhibited reduced bacterial adhesion compared to surfaces with microchannels that had a hydrophilic nature.

Of late, the fabrication of hierarchical surface structures using lasers has gained great importance. Christoph Zwahr et al.^85^ fabricated hierarchical surface structures on pure titanium metal by patterning microholes (by Direct Laser Interference Patterning) on top of large-sized microcraters (by Direct Laser Writing). These hierarchical structures showed a significant reduction in wear and comparatively decreased adhesion of the *E. coli* bacteria. Similar results have been reported by Gupta et al.,^78^ in which the hierarchical surface structures fabricated on titanium alloy by combining LIPSS and microscale structures exhibited a significant percentage of damaged and even dead *E. coli* bacterial cells compared to the polished surface and surface with microscale structures. LIPSS were produced by Direct Laser Writing on top of the microscale structures created by Laser Surface Melting. However, further studies must be performed to validate the capability of hierarchical surface structures to provide antimicrobial properties, biocompatibility, and mechanical stability.

Even though femtosecond lasers are the most appropriate for reducing the melting and resolidification of the substrate surface, studies have also reported that surface modification of polymers using femtosecond lasers is quite challenging compared to metals and ceramics due to the resolidification of the surface debris, which largely affects the surface topography and quality. This issue was solved in a study done by Ouyang et al.^74^ through a short-term annealing treatment of the laser-patterned PMMA specimen. They generated 3D microstructures having sharp edges and smooth inner walls on PMMA by placing the laser-textured specimen in a blast drying oven at room temperature and gradually increasing the temperature at a rate of 6 °C/min. After holding it for some time, the samples were allowed to cool, thereby reducing the surface irregularities and redeposited particles. After femtosecond laser processing, an altered layer of PMMA chains with a decreased molecular weight is formed on the PMMA surface. During annealing, this modified layer melts, and the molten materials are leveled to obtain a smooth effect on the microstructures. In a recent study, Xu et al.^86^ introduced three different microstructures, such as microgroove, microhoneycomb, and microcomposite structures on the zirconia surface through laser texturing, and microbial adhesion was found to be higher on the microgroove textures compared to the other two surface textures. Besides this, some unfavorable structures, like concave and corner shapes, formed on the surface, which became a place for increased microbial attachment. Hence, it is noteworthy that optimization of patterning parameters is necessary to generate surface structures that would minimize microbial adhesion.

### 3.3 Roughness

The role of surface roughness in minimizing microbial adhesion and biofilm formation and promoting osseointegration has been extensively explored. Compared to conventional surface texturing methods, direct laser texturing is highly effective in producing surfaces with uniform roughness.^87^ Surface modification of titanium alloy, in a study, using various methods such as sandblasting, acid-etching, and Er, Cr: YSGG laser, claimed that the laser textured surface has the highest roughness compared to other methods.^88^ Lasers are capable of generating surfaces with average roughness ranging between several micrometers (Figure 9). In a recent study, laser-based surface modification of zirconia produced groove-like structures of different periodicities, in which the valley and pile-up portions of the grooves had microscale roughness and nanoscale roughness along some of the valleys.^89^ An increase in laser fluence and repetition rate corresponds to an increase in roughness, whereas an increase in scanning speed corresponds to a decrease in roughness.^90^ Surface roughness is usually quantified using parameters such as average roughness (*R*_a_) and root-mean-square roughness (*R*_q_).^52^

**Figure 9:**
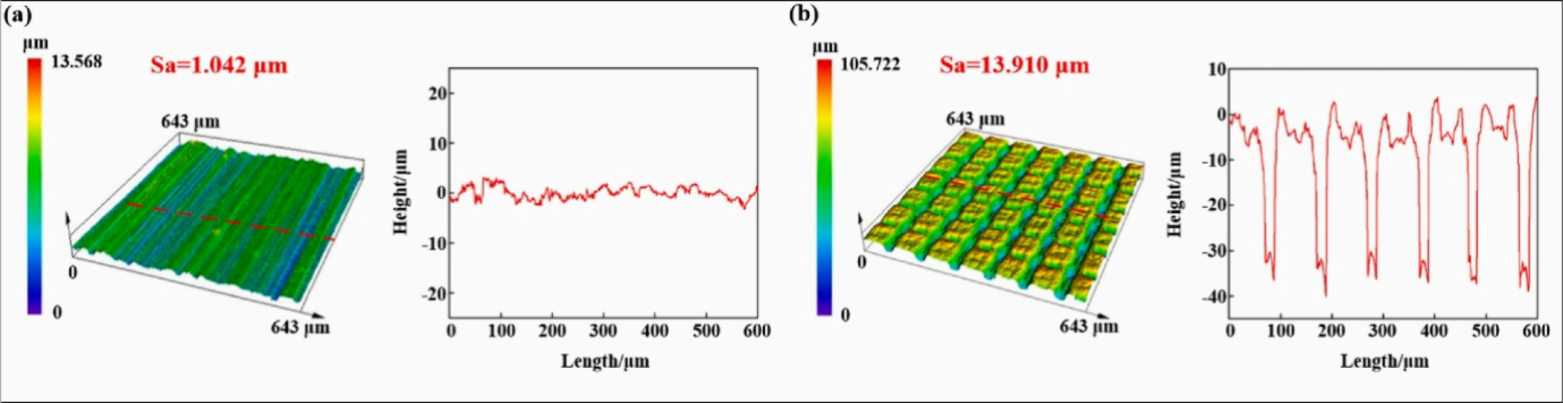
3D morphology and profile curve (roughness plot) of (a) untreated and (b) laser-treated zirconia ceramic surfaces analyzed by laser scanning confocal microscopy. Reprinted with permission from {Liu et al., Ceramics International} Copyright {2024} Elsevier.^91^

Several studies have shown that surface roughness on the nanoscale can reduce the adherence of microbes as the area of contact between the surface and the microbes decreases. In contrast, some other studies showed that the effect of the roughness on microbial adhesion was selective. An in vitro study reported that the Er, Cr: YSGG laser effectively reduced microbial adhesion on zirconia surfaces without causing significant alterations in surface microroughness.^92^ The observed variations in outcomes may be attributed to differences in the laser parameters used for surface texturing.

In addition, implant surface roughness has a significant effect on bone response to the implants.^93,94^ The average roughness (*R*_a_) of the biomaterial surface highly influences the healing period by anchoring cells and thus making a connection to the surrounding tissues. These surfaces show advantages over smooth polished surfaces as the surface area is increased through microstructuring of the material surface, and hence, more bone cells can attach to the surface.^95,96^ In other words, rough surfaces enhance cytocompatibility by promoting the adhesion of cells, proteins, and other growth factors.^97,98^

Meanwhile, observations of the relationship between the surface roughness and microbial adhesion are still controversial in the literature. Some studies showed that there is no significant reduction in microbial adhesion with increased surface roughness,^99^ while some other studies have attributed the reduction in bacterial proliferation to the high roughness up to 8 μm.^100^

In light of these observations from the existing literature, there is room for further investigation involving large-scale experiments on the effect of various laser patterning parameters on microbial adhesion. Additionally, modeling tools such as Taguchi methods can be used for prediction purposes.

### 3.4 Wettability

Wettability is an important surface property of dental biomaterials, and this property becomes crucial when it is connected to the prevention of microbial adhesion or the promotion of osseointegration. An ideal dental biomaterial requires a highly wettable surface and superior antibacterial properties. Increased wetness promotes tissue adhesion and growth on the material surface, thus reducing microbial adherence. In biological systems, wettability plays a pivotal role in increasing the interaction between solid and liquid surfaces.^101^ The surface wettability of materials is usually expressed in terms of hydrophilicity or hydrophobicity, which can be determined by analyzing the angle of contact of a liquid with the material surface (Figure 10). The material surface is hydrophilic if the contact angle of a liquid with the surface is less than 90° and hydrophobic if the contact angle is greater than 90°.^102^

**Figure 10:**
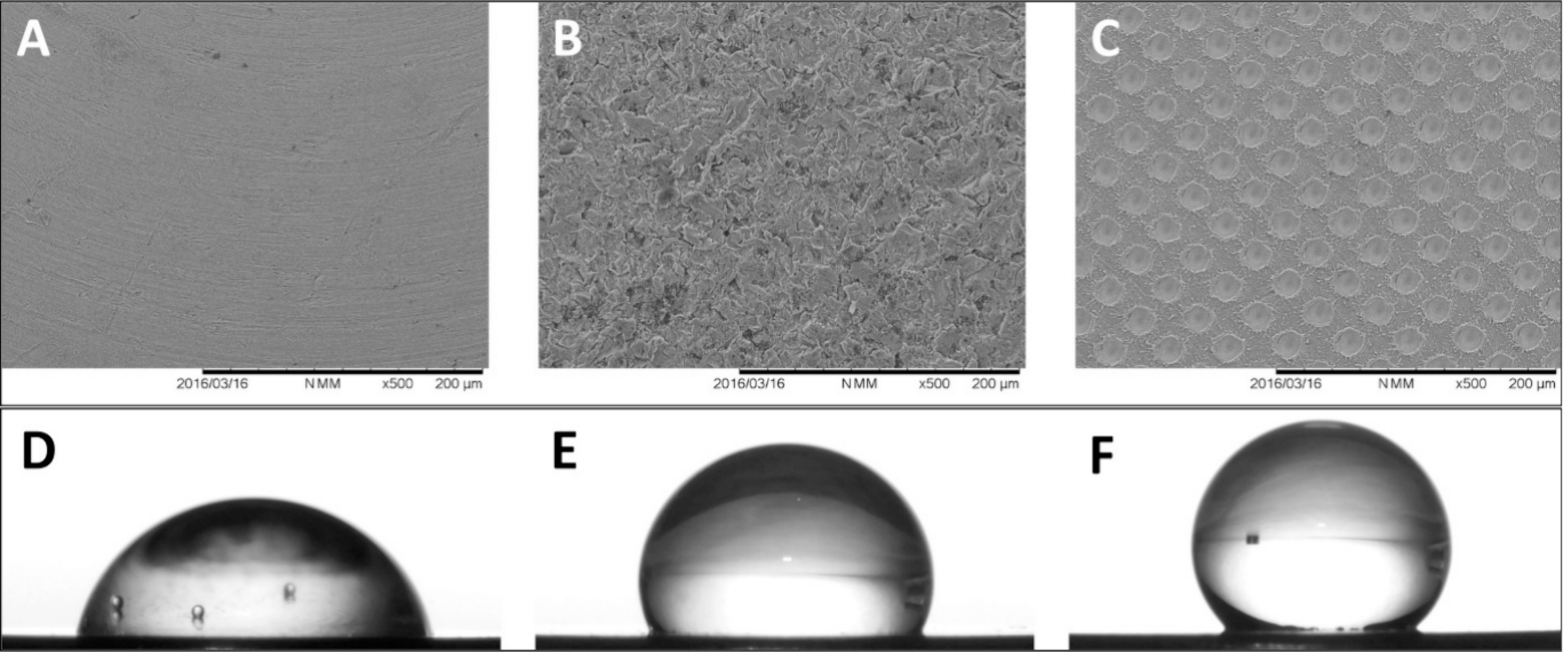
SEM images of (A) untreated, (B) grit-blasted, and (C) laser-treated titanium surfaces and (D–F) the corresponding images of a 6 μL drop of HPLC-grade ultrapure water on these surfaces, respectively. Reprinted with permission from {Ionescu et al., PLoS ONE} Copyright {2018} Public Library of Science.^103^

The structure and dimensions of the micro- or nanosized protrusions on the surface of materials greatly influence the wettability, thus behaving either as a hydrophilic or hydrophobic material.^104^ It was reported that laser-generated micrometer-scale cones on top of the submicrometer-scale ripples on titanium alloy were found beneficial in trapping air between the structures, which acted as a barrier to the penetration of water droplets into the surface structures. These surfaces exhibited superhydrophobicity compared to the LIPSS surface structures fabricated on the same material.^71^

Biomaterial surfaces that become easily wetted help enhance cell adhesion, differentiation, and proliferation during the initial stages of bone formation. Hydrophilic surfaces improve biological interactions, especially with blood. Laser patterning on commercially pure titanium samples has been reported to generate hydrophilic surfaces due to the complex surface nanostructures along with the presence of oxides, which improved the osseointegration of human mesenchymal stem cells.^105^ Moreover, studies have reported that femtosecond laser treatment can modify titanium alloy surfaces, thereby generating superhydrophobic surfaces, which reduced the fibrin network formation compared to the acid-etched surface that exhibited hydrophilicity.^106^ Hence, better surface wettability makes tissue attachment and integration better.

Recent research showed that femtosecond laser-induced surface modification of a bioactive glass material increased its wettability and enhanced its antibacterial property by minimizing the adhesion of three types of nosocomial bacterial species. Along with this, they also confirmed that laser texturing retained the biocompatibility of the material, as they observed that the attachment and growth of INT407 human cells remained unaltered compared to the untreated materials.^100^

Femtosecond laser-assisted modification has been reported to successfully alter the surfaces of titanium alloy, stainless steel, and zirconium-based bulk metallic glass by fabricating LIPSS, making them more hydrophilic than the control.^107^ XPS analysis of these material surfaces confirmed the presence of surface oxides after laser texturing, which increased the surface energy of the materials, thereby increasing the hydrophilicity. Even though the surface was hydrophilic, there was a significant reduction in the formation of biofilm and bacterial growth.^107^ A justification for this was given by Shaik et al.^84^ Their study regarding the surface treatment of titanium alloy (Ti6Al4 V) with a femtosecond laser to reduce bacterial adhesion reported that the sample surface showed very low contact angle values soon after laser patterning due to surface oxidation during laser irradiation; hence, the contact angle measurement was taken after several days and noted an increase in contact angle value from 73 to 160° when measured after 12 days. The transformation of the laser-textured surface from hydrophilic to superhydrophobic state can be attributed to changes in surface chemical composition and adsorption of carbon and other organic compounds from the atmosphere.^108^ Patil et al.^109^ utilized a thermal annealing treatment on the laser-textured grade-5 Ti–6Al–4 V alloy to accelerate the conversion of the surface from a hydrophilic to superhydrophobic state. Metal specimen surfaces will be hydrophilic immediately after laser treatment as a result of the formation of a thin, unstable metal oxide layer on the laser-textured specimen surface created as the molten metal reacts with atmospheric oxygen during ablation. Over 15 to 20 days, this oxide layer naturally thickens and stabilizes, and hence, thermal annealing of the laser-textured specimen surface would reduce the time for the transition to a superhydrophobic surface by accelerating the oxidation process, thereby increasing the chemical stability of the material surface. It is interesting to note that such surface changes may vary among the materials, depending on their chemical stability or inertness. In this regard, even though the polymeric surfaces are generally considered inert or less reactive, the effect of time duration on the hydrophilic or hydrophobic behavior is not fully explored.

Even though most of the studies proved that hydrophobic surfaces significantly reduce microbial adhesion, some studies showed that the antimicrobial effect can be generated by highly hydrophilic surfaces as well.^110^ Since the superhydrophilic (high surface energy) surfaces are surrounded by a monolayer of mobile water molecules, these water molecules cannot be displaced by any proteins or cells. Thus, such superhydrophilic surfaces prevent the adhesion of both cells and microbes on the surface, as the proteins will not directly interact with the surface and are anchored only through hydrogen bonds. However, these surfaces may not be appropriate for applications that require osseointegration, as they prevent the adhesion of cells.^111^

As mentioned in the Introductory section, the majority of the research considered material surface topography, roughness, and wettability as the prime factors influencing microbial adhesion. These three parameters are interrelated, too. Varying one parameter results in variation of the other two parameters. As an example, some recent studies on laser-assisted surface modification of dental biomaterials with the patterning parameters, observed surface characteristics, and their effect on microbial adhesion or osseointegration are represented in Table 3.^112–120^ It is worth noting that interpreting the actual effect of surface topography and wettability on microbial adhesion and osseointegration turns out to give contradictory conclusions most often, as surface roughness is considered to be the primary factor that describes both surface topography and wettability in a majority of studies. Moreover, such studies neglect the role of other characteristics, such as the chemical composition of the substrate material, its surface charge, stiffness, nature, and properties of the microbes and cells chosen for the assays. Even though these characteristics do not have much implication in controlling the rate of adhesion of microbes as well as bone cells compared with that of surface topography, roughness, and wettability, it is necessary to consider them as well. In this regard, it is important to analyze the surface properties from various aspects, so that the existing theories and results can be properly validated.

**Table 3:**
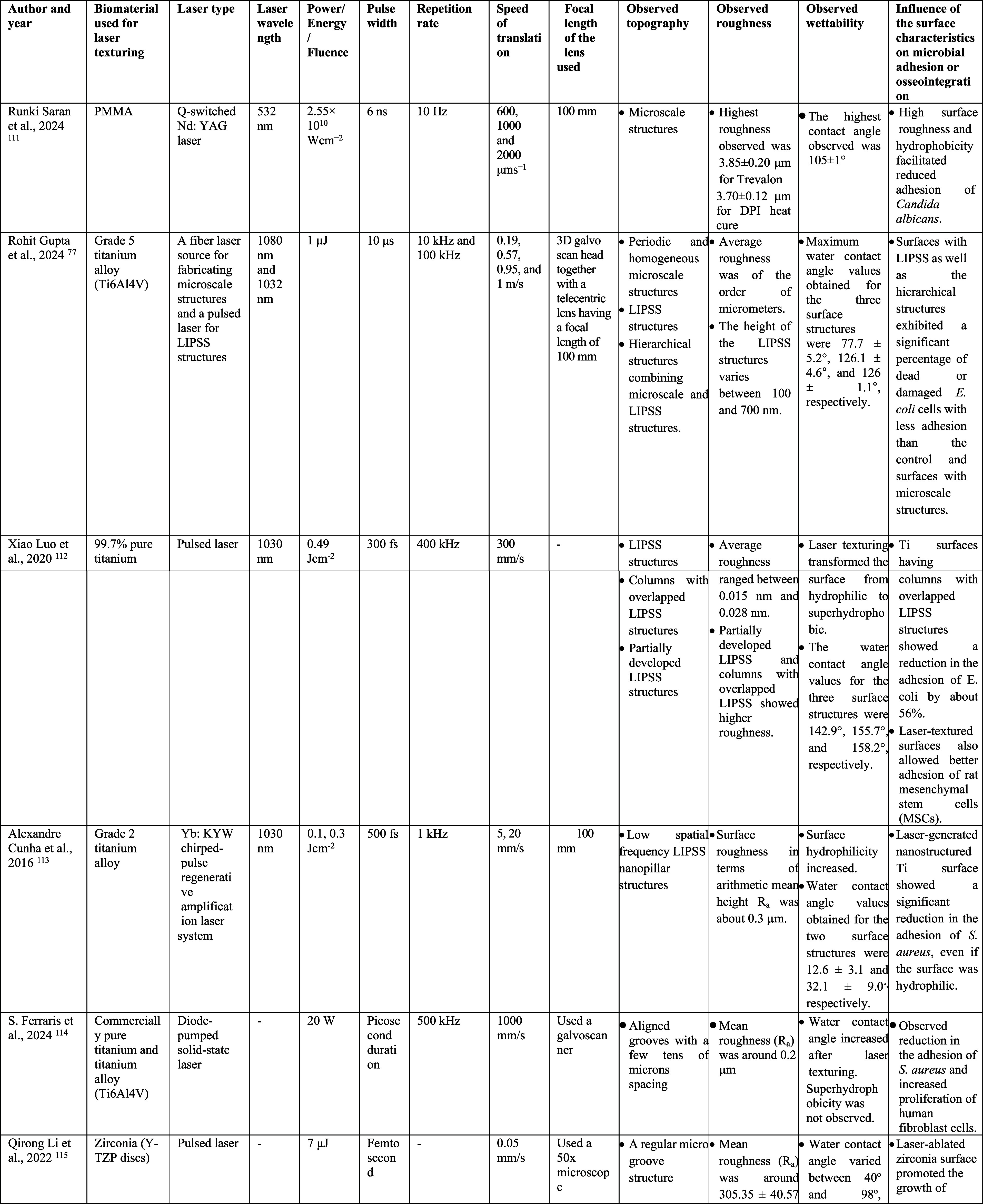

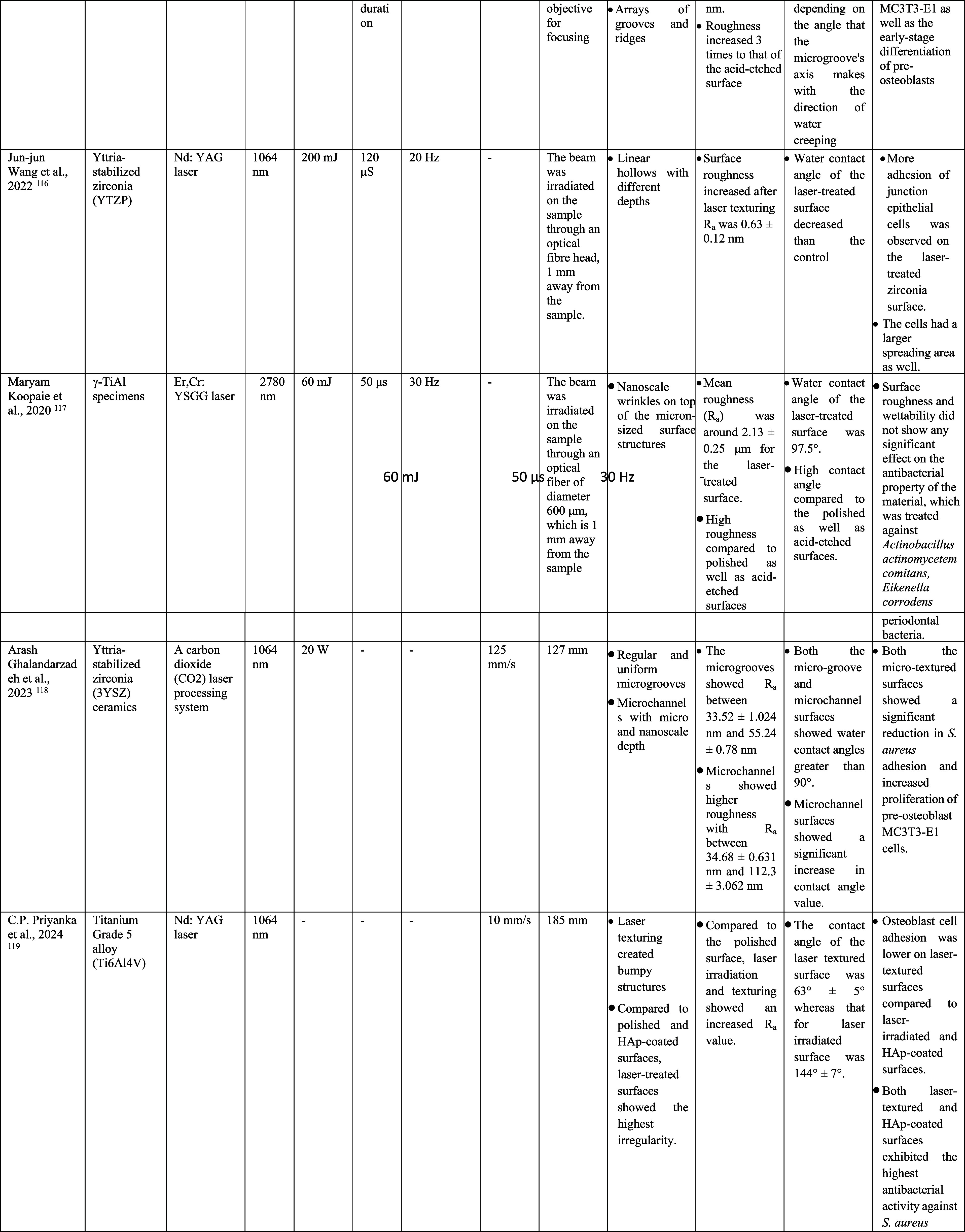
Some Recent Studies on Laser-Assisted Surface Modification of Dental Biomaterials with the Patterning Parameters, Observed Surface Characteristics, and Their Effect on Microbial Adhesion or Osseointegration

## 4 Conclusion and Future Trends

Dental biomaterials and related research have evolved considerably over the past 20 years, with their applicability progressively enhancing. Of late, lasers are utilized extensively for the surface modification of dental biomaterials. Laser-assisted surface texturing is a highly controlled and precise texturing method that can process various types of substrate materials, such as metals and alloys, ceramics, polymers, etc. By focusing a highly intense laser beam on the substrate material of interest, micro- and even nanoscale surface structures can be fabricated. This enhances the material’s surface properties like roughness and wettability, which in turn affect the cell response to the biomaterial as well as microbial adhesion. Studies have shown that laser-assisted surface texturing is capable of enhancing material characteristics and thereby reducing microbial adhesion as well as promoting osseointegration compared to conventional surface modification methods. The present review summarizes the utilization of lasers for the surface modification of dental biomaterials to promote better osseointegration and minimize microbial adhesion. Laser patterning parameters play a major role in modifying the surface topography, surface roughness, and wettability of a material. The major inferences from the research articles considered in this review are summarized in Table 4.

**Table 4:** Common Findings from the Studies Related to Laser-Assisted Surface Modification of Dental Biomaterials That Are Considered in This Review

parameter	inference
Laser parameters	• Pulsed lasers are commonly used with pulse widths ranging from nanoseconds, picoseconds, and femtoseconds. The majority of the recent research preferred femtosecond lasers.
	• Laser wavelengths adopted were mostly 355, 532, 800, 1030, and 1064 nm.
	• Laser energy or fluence varied depending on the ablation threshold of the materials considered for laser texturing in the respective study.
	• About 43% of the studies considered in this review adopted a repetition rate in the range of 1 to 1000 kHz for laser texturing (laser pulse durations, in such cases, were in the range of picoseconds or femtoseconds). A few studies adopted a repetition rate in the range of 1 to 100 Hz (laser pulse durations were in the nanosecond range).
Focusing optics	• Biconvex lenses of focal lengths 50, 100, and 200 mm were generally used for focusing the laser beam on the sample material. Some studies used objective lenses as well, which will offer better light collection and focusing, resulting in a smaller spot size.
	• A few studies that adopted a galvanometer scanner system used an f-theta lens of focal lengths 56 or 100 mm for focusing. A galvo scanner combined with f-theta lenses enables higher scanning speeds while ensuring consistent and uniform laser ablation across large surface areas.
Scanning speed of the translation stage	• Speed of translation (repetition rate of the laser as well) determines the distance between the laser-ablated craters. The space between the craters will be high if the scanning speed is high.
	• Scanning speed adopted varied in the range of 1 to 3000 mm/s.
Sample material used	• Around 68% of the studies included in this review pertain to laser-assisted surface modification of titanium, whereas research on polymer-based materials remains relatively limited.
	• Substrate surfaces subjected to laser texturing are generally flat. A few studies have conducted laser texturing on dental implant material having irregular surfaces. Surface patterning on irregular surfaces is quite challenging.
Microbes tested	• Evaluation of microbial adhesion was tested against *S. aureus, S. mutans, E. coli, C. albicans*, etc., which are the commonly seen microorganisms in the oral cavity.
Biological tissues tested for cytocompatibility	• Cell adhesion tests were carried out to investigate the adhesion and proliferation of cells such as human mesenchymal stem cells (hMSCs), human osteoblast-like osteosarcoma cells (MG-63), human gingival fibroblast cells, etc.

As the main essence of laser-based surface texturing is to modify the surface features of dental biomaterials, the fabrication of surface features with specific shapes and dimensions, respectively, to specific applications becomes crucial. Further studies are warranted to optimize the laser patterning parameters for any surface modification system that serves a specific function. In addition, there is a huge scope for further investigations of the longevity of biomaterials in terms of better osseointegration as well as reduced microbial adhesion. Despite extensive efforts by numerous researchers to clarify the underlying mechanisms governing the interaction between ultrafast laser irradiation and matter, a complete understanding of the same has not yet been achieved. Even though a wide range of ultrafast laser systems are available, scaling up the production volume continues to pose a significant challenge for clinical translation. Consequently, ongoing research and development are essential, serving not only fundamental scientific interests but also the advancement and commercialization of ultrafast laser processing technologies, especially for dental biomaterials. Moreover, hybrid approaches that combine additive and subtractive manufacturing techniques can enable greater diversity in geometry and enhanced functionality of fabricated structures, thereby further improving the overall performance of ultrafast laser processing of dental materials. Encouragingly, a majority of studies report favorable results, indicating significant enhancements achieved through laser texturing. Even though the results are promising, further research involving animal and human studies is essential to fully harness the potential of laser-surface texturing in enhancing the functionality of biomaterials. Comprehensive long-term clinical evidence remains limited, as there are very few in vivo studies being conducted, emphasizing the necessity for continued research to advance and refine these processes. Additionally, with the integration of artificial intelligence for the optimization of laser processing parameters and improved processing strategies, laser-based surface modification represents the future of dental biomaterials, transforming both patient comfort and clinical efficiency for practitioners.

## References

[1] Tzanakakis E.-G. C., Skoulas E., Pepelassi E., Koidis P., Tzoutzas I. G. (2021). The Use
of Lasers in Dental Materials: A Review. Materials
(Basel).

[2] Perrotti, V. ; Piattelli, A. ; Quaranta, A. ; Gómez-Moreno, G. ; Iezzi, G. ; Shelton, R. 1-biocompatibility of dental biomaterials. In Biocompatibility of Dental Biomaterials; Woodhead: 2017; 1–7.

[3] Liaqat S., Qayyum H., Rafaqat Z., Qadir A., Fayyaz S., Khan A., Jabeen H., Muhammad N., Khan M. A. (2022). Laser as
an innovative tool, its implications and advances in dentistry: A
systematic review. J. Photochem. Photobiol..

[4] Rolek A., Pławecki P. (2024). Advancements
and applications of laser technology in
modern dentistry. Wiad. Lek..

[5] Parker S. (2007). Introduction,
history of lasers and laser light production. British Dental Journal.

[6] Lagunov V. L., Rybachuk M., Itthagarun A., Walsh L. J., George R. (2022). Modification
of dental enamel, dentin by an ultra-fast femtosecond laser irradiation:
A systematic review. Optics & Laser Technology.

[7] Sugioka K. (2017). Progress in
ultrafast laser processing and future prospects. Nanophotonics.

[8] Lei S., Zhao X., Yu X., Hu A., Vukelic S., Jun M. B. G., Joe H.-E., Yao Y. L., Shin Y. C. (2020). Ultrafast
Laser Applications in Manufacturing Processes: A State-of-the-Art
Review. J. Manuf. Sci. Eng..

[9] Saran R., Ginjupalli K., George S. D., Chidangil S., V K U. (2023). LASER as a tool for surface modification of dental biomaterials:
A review. Heliyon.

[10] Sharma S., Ravi Kiran S., Kumar P., Shankar R., Kumar Upadhyay A. (2024). Per-Ingvar
Brånemark (1929–2014): A Homage to the Father of Osseointegration
and Modern Dentistry. Cureus.

[11] Laoui T., Santos E., Osakada K., Shiomi M., Morita M., Shaik S. K., Tolochko N. K., Abe F., Takahashi M. (2006). Properties
of Titanium Dental Implant Models Made by Laser Processing. Proc. Inst. Mech. Eng., C: J. Mech. Eng. Sci..

[12] Albrektsson T., Johansson C. (2001). Osteoinduction,
osteoconduction and osseointegration. Eur. Spine
J..

[13] Moraschini V., Poubel L. A. d. C., Ferreira V. F., Barboza E. d. S. P. (2015). Evaluation
of survival and success rates of dental implants reported in longitudinal
studies with a follow-up period of at least 10 years: a systematic
review. International Journal of Oral and Maxillofacial
Surgery.

[14] Zafar, M. S. ; Ullah, R. ; Qamar, Z. ; Fareed, M. A. ; Amin, F. ; Khurshid, Z. ; Sefat, F. Properties of dental biomaterials. In Advanced dental biomaterials; Elsevier: 2019; 7–35.

[15] Zierden K., Koch C. J., Wöstmann B., Rehmann P. (2024). Clinical Longevity
of Obturators in Patients with Jaw Defects: a Retrospective Cohort
Study. Clin. Oral Investig..

[16] Ferraris S., Spriano S. (2016). Antibacterial titanium surfaces for
medical implants. Materials Science and Engineering:
C.

[17] Dhayakaran, R. ; Neethirajan, S. Microscopic methods in biofilm research. In Biofilms: Emerging Concepts and Trends; Murthy, S. Ed.; Narosa Publishers: İndia, 2017.

[18] Liu B. H., Yu L.-C. (2017). In-situ, time-lapse study of extracellular
polymeric substance discharge
in Streptococcus mutans biofilm. Colloids Surf.,
B.

[19] Kreve S., Reis A. C. D. (2021). Bacterial adhesion
to biomaterials: What regulates
this attachment? A review. Japanese Dental Science
Review.

[20] Han A., Tsoi J. K. H., Rodrigues F. P., Leprince J. G., Palin W. M. (2016). Bacterial
adhesion mechanisms on dental implant surfaces and the influencing
factors. Int. J. Adhes. Adhes..

[21] Muhammad M. H., Idris A. L., Fan X., Guo Y., Yu Y., Jin X., Qiu J., Guan X., Huang T. (2020). Beyond Risk: Bacterial
Biofilms and Their Regulating Approaches. Front.
Microbiol..

[22] Filipović U., Dahmane R. G., Ghannouchi S., Zore A., Bohinc K. (2020). Bacterial
adhesion on orthopedic implants. Adv. Colloid
Interface Sci..

[23] Garaicoa J. L., Bates A. M., Avila-Ortiz G., Brogden K. A. (2020). Antimicrobial Prosthetic
Surfaces in the Oral Cavity-A Perspective on Creative Approaches. Microorganisms.

[24] Gheisarifar M., Thompson G. A., Drago C., Tabatabaei F., Rasoulianboroujeni M. (2021). In vitro study of surface alterations
to polyetheretherketone
and titanium and their effect upon human gingival fibroblasts. Journal of Prosthetic Dentistry.

[25] Rupp F., Liang L., Geis-Gerstorfer J., Scheideler L., Hüttig F. (2018). Surface characteristics of dental
implants: A review. Dental Materials.

[26] Andrukhov O., Huber R., Shi B., Berner S., Rausch-Fan X., Moritz A., Spencer N. D., Schedle A. (2016). Proliferation, behavior,
and differentiation of osteoblasts on surfaces of different microroughness. Dental Materials.

[27] Novaes A. B., de
Souza S. L. S., de Barros R. R. M., Pereira K. K. Y., Iezzi G., Piattelli A. (2010). Influence
of implant surfaces on osseointegration. Braz.
Dent. J..

[28] Barfeie A., Wilson J., Rees J. (2015). Implant surface
characteristics and
their effect on osseointegration. Br. Dent.
J..

[29] Zheng S., Bawazir M., Dhall A., Kim H.-E., He L., Heo J., Hwang G. (2021). Implication
of Surface Properties, Bacterial Motility,
and Hydrodynamic Conditions on Bacterial Surface Sensing and Their
Initial Adhesion. Front. Bioeng. Biotechnol..

[30] Zhu X., Jańczewski D., Guo S., Lee S. S. C., Parra
Velandia F. J., Teo S. L.-M., He T., Puniredd S. R., Vancso G. J. (2015). Polyion Multilayers with Precise Surface Charge Control
for Antifouling. ACS Appl. Mater. Interfaces.

[31] Wu S., Altenried S., Zogg A., Zuber F., Maniura-Weber K., Ren Q. (2018). Role of the surface nanoscale roughness of stainless steel on bacterial
adhesion and microcolony formation. ACS Omega.

[32] Helbig R., Günther D., Friedrichs J., Rößler F., Lasagni A., Werner C. (2016). The impact
of structure dimensions
on initial bacterial adhesion. Biomater. Sci..

[33] Wirth, J. ; Tahriri, M. ; Khoshroo, K. ; Rasoulianboroujeni, M. ; Dentino, A. R. ; Tayebi, L. , 6 - Surface modification of dental implants. In Biomaterials for Oral and Dental Tissue Engineering, Tayebi, L. ; Moharamzadeh, K. , Eds.; Woodhead Publishing: 2017; 85–96.

[34] Jemat A., Ghazali M. J., Razali M., Otsuka Y. (2015). Surface modifications
and their effects on titanium dental implants. Biomed. Res. Int..

[35] Smeets R., Stadlinger B., Schwarz F., Beck-Broichsitter B., Jung O., Precht C., Kloss F., Gröbe A., Heiland M., Ebker T. (2016). Impact of dental implant surface
modifications on osseointegration. Biomed. Res.
Int..

[36] Kligman S., Ren Z., Chung C.-H., Perillo M. A., Chang Y.-C., Koo H., Zheng Z., Li C. (2021). The Impact of Dental Implant Surface
Modifications on Osseointegration and Biofilm Formation. J. Clin. Med..

[37] Seo B. Y., Son K., Son Y.-T., Dahal R. H., Kim S., Kim J., Hwang J., Kwon S.-M., Lee J.-M., Lee K.-B., Kim J.-W. (2023). Influence
of Dental Titanium Implants with Different
Surface Treatments Using Femtosecond and Nanosecond Lasers on Biofilm
Formation. J. Funct. Biomater..

[38] Prodanov L., Lamers E., Wolke J., Huiberts R., Jansen J. A., Frank Walboomers X. (2014). In vivo comparison between laser-treated and grit blasted/acid
etched titanium. Clin. Oral Implants Res..

[39] Spriano S., Yamaguchi S., Baino F., Ferraris S. (2018). A critical review of
multifunctional titanium surfaces: New frontiers for improving osseointegration
and host response, avoiding bacteria contamination. Acta Biomaterialia.

[40] Ma C., Peng G., Nie L., Liu H., Guan Y. (2018). Laser surface
modification of Mg-Gd-Ca alloy for corrosion resistance and biocompatibility
enhancement. Appl. Surf. Sci..

[41] Faeda R. S., Tavares H. S., Sartori R., Guastaldi A. C., Marcantonio E. (2009). Evaluation of
titanium implants with
surface modification by laser beam: biomechanical study in rabbit
tibias. Braz. Oral Res..

[42] Brånemark R., Emanuelsson L., Palmquist A., Thomsen P. (2011). Bone response to laser-induced
micro-and nano-size titanium surface features. Nanomedicine.

[43] Ukar E., Lamikiz A., Martínez S., Arrizubieta I. (2015). Laser texturing
with conventional fiber laser. Procedia Eng..

[44] Wang H., Deng D., Zhai Z., Yao Y. (2024). Laser-processed functional
surface structures for multi-functional applications-a review. J. Manuf. Process..

[45] Shabir S., Khan N. Z., Siddiquee A. (2025). Laser-induced
surface modifications
in biomedical materials: a comprehensive review. Surf. Topogr.: Metrol. Prop..

[46] Yap C. Y., Chua C. K., Dong Z. L., Liu Z. H., Zhang D. Q., Loh L. E., Sing S. (2015). Review of
selective laser melting:
Materials and applications. Appl. Phys. Rev..

[47] Chrisey, D. B. ; Hubler, G. Pulsed laser deposition, 1994. 10.1097/00002480-199407000-00126 8555642

[48] Henriques B., Fabris D., Voisiat B., Boccaccini A. R., Lasagni A. F. (2024). Direct Laser Interference
Patterning of Zirconia Using
Infra-Red Picosecond Pulsed Laser: Effect of Laser Processing Parameters
on the Surface Topography and Microstructure. Adv. Funct. Mater..

[49] El-Khoury M., Voisiat B., Kunze T., Lasagni A. F. (2020). Prediction of Optimum
Process Parameters Fabricated by Direct Laser Interference Patterning
Based on Central Composite Design. Materials
(Basel).

[51] Bleu Y., Bourquard F., Tite T., Loir A.-S., Maddi C., Donnet C., Garrelie F. (2018). Review of graphene growth from a
solid carbon source by pulsed laser deposition (PLD). Front. Chem..

[52] Chu S.-F., Huang M.-T., Ou K.-L., Sugiatno E., Cheng H.-Y., Huang Y.-H., Chiu W.-T., Liou T.-H. (2016). Compounds, Enhanced
biocompatible and hemocompatible nano/micro porous surface as a biological
scaffold for functionalizational and biointegrated implants. J. Alloys Compd..

[53] Rethfeld B., Ivanov D. S., Garcia M. E., Anisimov S. I. (2017). Modelling ultrafast
laser ablation. J. Phys. D: Appl. Phys..

[54] Liu X., Du D., Mourou G. (1997). Laser ablation
and micromachining with ultrashort laser
pulses. IEEE J. Quantum Electron..

[55] Knowles M.
R. H., Rutterford G., Karnakis D., Ferguson A. (2007). Micro-machining of
metals, ceramics and polymers using nanosecond lasers. International Journal of Advanced Manufacturing Technology.

[56] Wahab J. A., Ghazali M. J., Yusoff W. M. W., Sajuri Z. (2016). Enhancing material
performance through laser surface texturing: a review. Transactions of the IMF.

[57] Pou-Álvarez P., Riveiro A., Nóvoa X. R., Fernández-Arias M., del Val J., Comesaña R., Boutinguiza M., Lusquiños F., Pou J. (2021). Nanosecond, picosecond
and femtosecond
laser surface treatment of magnesium alloy: role of pulse length. Surf. Coat. Technol..

[58] Chichkov B. N., Momma C., Nolte S., von Alvensleben F., Tünnermann A. (1996). Femtosecond, picosecond and nanosecond
laser ablation
of solids. Appl. Phys. A: Mater. Sci. Process..

[59] Wang S., Yang J., Deng G., Zhou S. (2024). Femtosecond laser direct
writing of flexible electronic devices: A mini review. Materials.

[60] Shugaev M. V., Wu C., Armbruster O., Naghilou A., Brouwer N., Ivanov D. S., Derrien T. J. Y., Bulgakova N. M., Kautek W., Rethfeld B., Zhigilei L. V. (2016). Fundamentals
of ultrafast laser–material interaction. MRS Bull..

[61] Lin Z., Hong M. (2021). Femtosecond Laser Precision Engineering: From Micron, Submicron,
to Nanoscale. Ultrafast Sci..

[62] Molian P., Pecholt B., Gupta S. (2009). Picosecond pulsed laser ablation
and micromachining of 4H-SiC wafers. Appl. Surf.
Sci..

[63] Rethfeld B., Sokolowski-Tinten K., Von Der Linde D., Anisimov S. (2004). Timescales in the response
of materials to femtosecond laser excitation. Appl. Phys..

[64] Mao B., Siddaiah A., Liao Y., Menezes P. L. (2020). Laser surface texturing
and related techniques for enhancing tribological performance of engineering
materials: A review. Journal of Manufacturing
Processes.

[65] Gao H., Wang Y., Ye J., Du B., Wang D., Li S., Cui Q., Wang S., Zhang T. (2025). Study on the Ablation
Behavior of High-Intensity Lasers in Vacuum. Appl. Sci..

[66] Zhou H., Li C., Zhou Z., Cao R., Chen Y., Zhang S., Wang G., Xiao S., Li Z., Xiao P. (2018). Femtosecond
laser-induced periodic surface microstructure on dental zirconia ceramic. Mater. Lett..

[67] Trtica M., Gakovic B., Batani D., Desai T., Panjan P., Radak B. (2006). Surface modifications of a titanium
implant by a picosecond Nd:YAG
laser operating at 1064 and 532nm. Appl. Surf.
Sci..

[68] Sun J., Yang Y., Wang D. (2013). Parametric optimization of selective
laser melting for forming Ti6Al4V samples by Taguchi method. Optics & Laser Technology.

[69] Wang X., Duan J., Jiang M., Zhang F., Ke S., Wu B., Zeng X. (2017). Investigation
of processing parameters for three-dimensional
laser ablation based on Taguchi method. International
Journal of Advanced Manufacturing Technology.

[70] Imran H. J., Hubeatir K. A., Al-Khafaji M. M. (2021). CO2 Laser
Micro-Engraving of PMMA
Complemented By Taguchi and ANOVA Methods. Journal
of Physics: Conference Series.

[71] Lu L., Zhang J., Guan K., Zhou J., Yuan F., Guan Y. (2022). Artificial neural network
for cytocompatibility and antibacterial
enhancement induced by femtosecond laser micro/nano structures. J. Nanobiotechnol..

[72] Uhlmann E., Schweitzer L., Kieburg H., Spielvogel A., Huth-Herms K. (2018). The Effects
of Laser Microtexturing of Biomedical Grade
5 Ti-6Al-4V Dental Implants (Abutment) on Biofilm Formation. Procedia CIRP.

[73] Nathala C. S., Ajami A., Ionin A. A., Kudryashov S. I., Makarov S. V., Ganz T., Assion A., Husinsky W. (2015). Experimental
study of fs-laser induced sub-100-nm periodic surface structures on
titanium. Opt. Express.

[74] Ouyang Z., Long J., Wu J., Lin J., Xie X., Tan G., Yi X. (2022). Preparation of high-quality three-dimensional
microstructures
on polymethyl methacrylate surfaces by femtosecond laser micromachining
and thermal-induced micro-leveling. Optics &
Laser Technology.

[75] Sunderlal
Singh S., Samuel G. L. (2024). Near-infrared femtosecond laser direct
writing of microchannel and controlled surface wettability. Optics & Laser Technology.

[76] Patel D. S., Singh A., Balani K., Ramkumar J. J. S. (2018). Topographical
effects of laser surface texturing on various time-dependent wetting
regimes in Ti6Al4V. Surf. Coat. Technol..

[77] Zhao L., Wang Z., Zhang J., Cao L., Li L., Yue Y., Li D. (2015). Antireflection silicon
structures with hydrophobic
property fabricated by three-beam laser interference. Appl. Surf. Sci..

[78] Gupta R., Gaddam A., Prajapati D., Dimov S., Mishra A., Vadali M. (2024). Enhancing Bactericidal
Properties of Ti6Al4V Surfaces
through Micro and Nano Hierarchical Laser Texturing. Langmuir.

[79] Gnilitskyi I., Pogorielov M., Viter R., Ferraria A. M., Carapeto A. P., Oleshko O., Orazi L., Mishchenko O. (2019). Cell and tissue
response to nanotextured Ti6Al4V and Zr implants using high-speed
femtosecond laser-induced periodic surface structures. Nanomedicine.

[80] Orazi L., Pelaccia R., Mishchenko O., Reggiani B., Pogorielov M. (2020). Fast LIPSS
based texturing process of dental implants with complex geometries. CIRP Annals.

[81] Gnilitskyi I., Dolgov L., Tamm A., Ferraria A. M., Diedkova K., Kopanchuk S., Tsekhmister Y., Veiksina S., Polewczyk V., Pogorielov M. (2024). Enhanced osteointegration
and osteogenesis of osteoblast
cells by laser-induced surface modification of Ti implants. Nanomedicine: Nanotechnology, Biology and Medicine.

[82] Deppe H., Warmuth S., Heinrich A., Körner T. (2005). Laser-assisted
three-dimensional surface modifications of titanium implants: preliminary
data. Lasers in Medical Science.

[83] Heinrich A., Dengler K., Koerner T., Haczek C., Deppe H., Stritzker B. (2007). Laser-modified
titanium implants for improved cell
adhesion. Lasers in Medical Science.

[84] Shaikh S., Kedia S., Singh D., Subramanian M., Sinha S. (2019). Surface texturing of Ti6Al4V alloy
using femtosecond laser for superior
antibacterial performance. J. Laser Appl..

[85] Zwahr C., Helbig R., Werner C., Lasagni A. F. (2019). Fabrication of multifunctional
titanium surfaces by producing hierarchical surface patterns using
laser based ablation methods. Sci. Rep..

[86] Xu J., Ji M., Li L., Wu Y., Yu Q., Chen M. (2022). Improving
wettability, antibacterial and tribological behaviors of zirconia
ceramics through surface texturing. Ceram. Int..

[87] Saran R., Kanneeram K., Unnikrishnan V. K., Daniel George S., Ghurye A. A., Raghu
Chandrashekar H., Perampalli N. U., Ginjupalli K. (2025). Effect of
nanosecond laser assisted surface modification
on physical and mechanical properties of denture base materials. Sci. Rep..

[88] Abdulla M. A., Hasan R. H., Al-Hyani O. H. (2023). Impact
of Er,Cr:YSGG Laser, Sandblast,
and Acid Etching Surface Modification on Surface Topography of Biodental
Titanium Implants. J. Lasers Med. Sci..

[89] García
de Albéniz López de Aberasturi N., Roa Rovira J. J., Mas Moruno C. (2022). Laser-assisted surface modification of zirconia-based
materials to guide osteoblast responses for dental applications. Material-ES.

[90] Braga F. J. C., Marques R. F. C., Filho E. d. A., Guastaldi A. C. (2007). Surface
modification of Ti dental implants by Nd:YVO4 laser irradiation. Appl. Surf. Sci..

[91] Liu C., Fu J., Li L., Wang H., Pei X., Zhang T., Wang Q. (2024). Fabrication
of antibacterial and anti-corrosive zirconia ceramics
with extreme wettability by facile laser-based surface modification. Ceram. Int..

[92] Pham C. M., Chen C.-Y., Kim D. M. (2021). The effects
of using erbium, chromium-doped:yttrium-scandium-gallium-garnet
laser on the surface modification, bacterial decontamination, and
cell adhesion on zirconia discs: an in vitro study. Lasers in Medical Science.

[93] Chikarakara E., Fitzpatrick P., Moore E., Levingstone T., Grehan L., Higginbotham C., Vázquez M., Bagga K., Naher S., Brabazon D. (2015). In vitro fibroblast
and pre-osteoblastic cellular responses on laser surface modified
Ti–6Al–4V. Biomedical Materials.

[94] Shiba T., Ho K., Ma X., Cho Y. W., Chen C.-Y., Kim D. M. (2024). Effect
of Er,Cr:YSGG Laser Irradiation on the Surface Modification and Cell
Adhesion on Titanium Discs: An In Vitro Study. Materials.

[95] Györgyey Á., Ungvári K., Kecskeméti G., Kopniczky J., Hopp B., Oszkó A., Pelsöczi I., Rakonczay Z., Nagy K., Turzó K. (2013). Attachment
and proliferation of human osteoblast-like cells (MG-63) on laser-ablated
titanium implant material. Materials Science
and Engineering: C.

[96] Bereznai M., Pelsöczi I., Tóth Z., Turzó K., Radnai M., Bor Z., Fazekas A. (2003). Surface modifications
induced by ns and sub-ps excimer laser pulses on titanium implant
material. Biomaterials.

[97] Gujba A. K., Medraj M. (2014). Laser Peening Process
and Its Impact on Materials Properties
in Comparison with Shot Peening and Ultrasonic Impact Peening. Materials (Basel).

[98] Yu Z., Yang G., Zhang W., Hu J. (2018). Investigating the effect
of picosecond laser texturing on microstructure and biofunctionalization
of titanium alloy. J. Mater. Process. Technol..

[99] Pacha-Olivenza M. Á., Tejero R., Fernández-Calderón M. C., Anitua E., Troya M., González-Martín M. (2019). Relevance
of topographic parameters on the adhesion and proliferation of human
gingival fibroblasts and oral bacterial strains. Biomed. Res. Int..

[100] Shaikh S., Singh D., Subramanian M., Kedia S., Singh A. K., Singh K., Gupta N., Sinha S. (2018). Femtosecond laser induced surface modification for prevention of
bacterial adhesion on 45S5 bioactive glass. J. Non-Cryst. Solids.

[101] Orazi L., Gnilitskyi I., Serro A. (2017). Laser nanopatterning
for wettability applications. J. Micro Nano-Manuf..

[102] Peethan, A. ; Unnikrishnan, V. ; Chidangil, S. ; George, S. ; Laser-Assisted Tailoring of Surface Wettability-Fundamentals and Applications: A Critical Review. Progress in Adhesion and Adhesives; Wiley, 2020, 5, 331–365.

[103] Ionescu A. C., Brambilla E., Azzola F., Ottobelli M., Pellegrini G., Francetti L. A. (2018). Laser microtextured titanium implant
surfaces reduce in vitro and in situ oral biofilm formation. PLoS One.

[104] Wang B., Zhang Y., Song J., Wang Z. (2020). Investigation
and Prediction on Regulation of Hydrophobicity of Polymethyl Methacrylate
(PMMA) Surface by Femtosecond Laser Irradiation. Coatings.

[105] Bressel T. A. B., de Queiroz J. D. F., Gomes Moreira S. M., da Fonseca J. T., Filho E. A., Guastaldi A. C., de Medeiros S. R. B. (2017). Laser-modified
titanium surfaces enhance the osteogenic
differentiation of human mesenchymal stem cells. Stem Cell Res. Ther..

[106] Lackington W. A., Schweizer P., Khokhlova M., Cancellieri C., Guimond S., Chopard-Lallier A.-L., Hofstetter J., Schmutz P., Maeder X., Rottmar M. (2022). Femtosecond
Laser-Texturing the Surface of Ti-Based Implants to Improve Their
Osseointegration Capacity. Adv. Mater. Interfaces.

[107] Du C., Wang C., Zhang T., Zheng L. (2022). Antibacterial Performance
of Zr-BMG, Stainless Steel, and Titanium Alloy with Laser-Induced
Periodic Surface Structures. ACS Applied Bio
Materials.

[108] Li P., Zhou T., Zhang M., Guo W., Cui Y., Zhang D., Liu P., Zhou L., Zhou X., He H. (2024). Surface texture fabricated by ultrafast laser treatment
for manipulating wettability and cell adhesion performance of Ti6Al4V. Surf. Coat. Technol..

[109] Patil D., Aravindan S., Wasson M. K., Perumal V., Paruchuri V. R. (2017). Fast Fabrication
of Superhydrophobic Titanium Alloy
as Antibacterial Surface Using Nanosecond Laser Texturing. J. Micro Nano-Manuf..

[110] Legeay G., Poncin-Epaillard F., Arciola C. (2006). New surfaces with hydrophilic/hydrophobic
characteristics in relation to (no) bioadhesion. Int. J. Artif. Organs.

[111] Norowski P. A., Bumgardner J. D. (2009). Biomaterial
and antibiotic strategies for peri-implantitis: A review. J. Biomed. Mater. Res. B Appl. Biomater..

[112] Saran R., K K., V K U., George S. D., H R. C., Upadhya P N., Ginjupalli K. (2024). Can pulsed
laser treatment reduce microbial adhesion on the surface of resin
denture base materials?. Materials Research
Express.

[113] Luo X., Yao S., Zhang H., Cai M., Liu W., Pan R., Chen C., Wang X., Wang L., Zhong M. (2020). Biocompatible
nano-ripples structured surfaces induced by femtosecond laser to rebel
bacterial colonization and biofilm formation. Optics & Laser Technology.

[114] Cunha A., Elie A.-M., Plawinski L., Serro A. P., Botelho do Rego A. M., Almeida A., Urdaci M. C., Durrieu M.-C., Vilar R. (2016). Femtosecond laser surface texturing
of titanium as a method to reduce the adhesion of Staphylococcus aureus
and biofilm formation. Appl. Surf. Sci..

[115] Ferraris S., Cochis A., Scalia A. C., Tori A., Rimondini L., Spriano S. (2024). Laser surface texturing
of Ti-cp
and Ti6Al4V alloy for the improvement of fibroblast adhesion and alignment
and the reduction of bacterial adhesion. Journal
of Materials Research and Technology.

[116] Grosgogeat B., Vaicelyte A., Gauthier R., Janssen C., Le Borgne M. (2022). Toxicological
Risks of the Cobalt–Chromium Alloys
in Dentistry: A Systematic Review. Materials.

[117] Wang J.-j., Zhang M., Wang Y.-j., Jia L.-n., Yang J.-j., Zhang Q. (2022). The effects of using Nd:YAG laser
on the surface modification and cell adhesion/spreading on zirconia
discs: An in vitro study. Mater. Lett..

[118] Koopaie M., Kia Darbandsari A., Hakimiha N., Kolahdooz S. (2020). Er,Cr:YSGG
laser surface treatment of gamma titanium aluminide: Scanning electron
microscopy–energy-dispersive X-ray spectrometer analysis, wettability
and Eikenella corrodens and Aggregatibacter actinomycetemcomitans
bacteria count—in vitro study. Proceedings
of the Institution of Mechanical Engineers, Part H: Journal of Engineering
in Medicine.

[119] Ghalandarzadeh A., Ganjali M., Hosseini M. (2023). Effects of
surface
topography through laser texturing on the surface characteristics
of zirconia-based dental materials: surface hydrophobicity, antibacterial
behavior, and cellular response. Surface Topography:
Metrology and Properties.

[120] Priyanka C. P., Sudeep U., Keerthi
Krishnan K., Ramachandran K. K. (2024). Surface characterisation and in vitro
osteogenic and
bacterial adhesion assays of laser treated and hydroxyapatite coated
Ti6Al4V bioimplant surfaces. Materials Today
Communications.

